# Gompertz growth with a shared carrying capacity optimally simulates primary and metastatic tumor growth dynamics

**DOI:** 10.1038/s41416-025-03306-9

**Published:** 2026-02-24

**Authors:** Pirmin Schlicke, Preethi Korangath, Xiaoxi Pan, Caner Ercan, Kathleen Gabrielson, Lyndsey Werhane, Yinyin Yuan, Sébastien Benzekry, Robert Ivkov, Heiko Enderling

**Affiliations:** 1 Department of Radiation Oncology, The University of Texas MD Anderson Cancer Center, Houston, TX USA; 2 Institute for Data Science in Oncology, The University of Texas MD Anderson Cancer Center, Houston, TX USA; 3 Department of Radiation Oncology and Radiation Sciences, Johns Hopkins University School of Medicine, Baltimore, ML USA; 4 Department of Translational Molecular Pathology, The University of Texas MD Anderson Cancer Center, Houston, TX USA; 5 Department of Molecular and Comparative Pathobiology, Johns Hopkins University School of Medicine, Baltimore, ML USA; 6 COMPutational Pharmacology and Clinical Oncology Department, Aix-Marseille University, Marseille, France; 7 Department of Oncology, Johns Hopkins University School of Medicine, Baltimore, ML USA; 8 Department of Mechanical Engineering, Johns Hopkins University, Baltimore, ML USA; 9 Department of Materials Science and Engineering, Whiting School of Engineering, Johns Hopkins University, Baltimore, ML USA; 10 Present Address: Department of Biosciences and Medical Biology, University of Salzburg, Salzburg, Austria

**Keywords:** Metastasis, Differential equations, Metastasis, Computational models

## Abstract

**Background:**

Cancer is a systemic disease with most deaths attributed to metastatic burden. Primary and metastatic tumors, albeit at different anatomic locations, are interconnected through multiple biological processes. Pre-clinical and clinical observations of growth acceleration of metastases after surgery, or abscopal effects outside the radiation field are widely reported, yet reliably triggering favorable and avoiding unfavorable systemic responses remains an unmet clinical need. Understanding local and systemic tumor interaction dynamics will help guide future treatments.

**Methods:**

We analyze the data of multiple in vivo tumor models. We formalize the systemic interplay of tumors as mathematical differential equation and calibrate parameters for each cell line and mouse type. Using model selection metrics, we identify classic tumor growth models with a novel shared carrying capacity parsimoniously describe the pan-cancer experimental data.

**Results:**

Shared systemic carrying capacity, metastatic spread potential, and metastatic growth rates differ across tested cell lines and mouse strains. Bi-directional concomitant systemic interconnectivity explains the observed metastatic explosion after primary tumor surgery.

**Discussion:**

Future investigations should reproduce this analysis in clinical settings and evaluate whether this shared carrying capacity model could help stratify patients at risk of metastatic disease below clinical detectability and inform strategies to control oligometastatic cancer.

## Quick guide to equations and assumptions

The Gompertz equation, or Gompertzian growth, is an established growth law for tumor volume over time [1]:

$$\frac{{dT}}{{dt}}={rT\; ln}\left(\frac{K}{T}\right)$$

where *T*(*t*) is the size of a tumor with unit [cc] at a given time *t* [day]. *r* [day^−1^] is the intrinsic tumor growth rate in an unconstrained condition, and *K* [cc] is the tumor carrying capacity. The tumor carrying capacity is the maximum tumor volume that can be attained by the current tumor microenvironment and other patient-specific conditions [2]. Together, ${rT}{\mathrm{ln}}\left(\frac{K}{T}\right)$ is the effective change in tumor size at time *t*.

The Gompertz equation has been shown to provide excellent fits to pre-clinical and clinical tumor growth data [3]. To date, it has only been applied to simulate a single tumor at a time. Here, we introduce a system of two coupled Gompertzian equations for two tumors, *T*_1_ and *T*_2_, growing simultaneously in the same host (mouse or patient) with their intrinsic growth rates *r*_1_ and *r*_2_, and a shared carrying capacity *K*, such that *K* describes the maximum tumor volume that can be obtained by the sum of *T*_1_ and *T*_2_:

$$\frac{d{T}_{1}}{{dt}}={r}_{1}{T}_{1}\left(t\right)\,{ln}\left(\frac{K}{{T}_{1}\left(t\right)+{T}_{2}\left(t\right)}\right){{\rm{and}}}\frac{d{T}_{2}}{{dt}}={r}_{2}{T}_{2}\left(t\right)\,{ln}\left(\frac{K}{{T}_{1}\left(t\right)+{T}_{2}\left(t\right)}\right)$$

This coupled system examines the overall interconnected growth dynamics of two tumors, which can be compared against a model of two independently growing tumors with individual carrying capacities *K*_1_ and *K*_2_.

$$\frac{d{T}_{1}}{{dt}}={r}_{1}{T}_{1}\left(t\right)\,{ln}\left(\frac{{K}_{1}}{{T}_{1}\left(t\right)}\right){{\rm{and}}}\frac{d{T}_{2}}{{dt}}={r}_{2}{T}_{2}\left(t\right)\,{ln}\left(\frac{{K}_{2}}{{T}_{2}\left(t\right)}\right)$$

This concept can be further advanced to simulated metastatic seeding and systemic interconnectivity between the primary tumor and its metastatic offspring through a shared carrying capacity.

Let $\rho (x,t)$ denote the density distribution of the metastases of size *x* at time *t*, the Gompertzian term is replaced with ${\mathrm{ln}}\left(\frac{{K}_{1}}{\theta \left(t\right)}\right)$ where *θ*(t) is the time-specific overall tumor load with

$$\theta \left(t\right)=\,{x}_{{PT}}\left(t\right)+\,\int ^{\infty }_{1}\widetilde{x}\rho (\widetilde{x},t)d\widetilde{x}.$$

Here, *x*_*PT*_ denotes the primary tumor and $\widetilde{x}$ the individual metastases. The mathematical details of metastatic seeding from the primary tumor and second order seeding from metastases are shown in the Materials and Methods section.

## Introduction

Metastatic cancer is a systemic disease [4], and nine out of ten cancer-related deaths are due to the presence of metastases [5]. Different clinical outcomes are directly related to the behavior of circulating tumor cells, metastatic tumors and their systemic interplay with the primary tumor [6–8]. Therefore, understanding the interactions between the primary and metastatic tumors, their effects and changes of these under certain treatments are highly relevant and important for treatment considerations and successful patient management.

‘Concomitant resistance’, the inhibition of secondary tumor growth by the presence of a primary tumor, was first described by Paul Ehrlich [9, 10] in the 1900s, and subsequently demonstrated in both animal models and clinical studies [11]. Pioneering studies focused on “concomitant immunity” [12] where a primary tumor generates an immune response to inhibit metastatic growth through suppression or activation of antitumor effects of CD8 + T cells [13–15]. However, concomitant resistance has been observed in immunogenic and T cell-defective tumors alike, rejecting initial assumptions that this phenomenon is solely driven by T cell-mediated immune interactions between primary and secondary tumors [16–19]. More recent mechanisms contributing to concomitant resistance include tumor-induced release of molecules that inhibit growth and promote dormancy of metastatic cells; and athrepsia, the growth inhibition of one tumor through another one due to competition for essential host factors such as resources and space [11, 20–22].

Clinical observations supporting systemic interconnectivity of multiple tumors include accelerated metastatic growth after surgical removal of the primary tumor [16, 23, 24] as well as the regression of distant, non-irradiated metastases after primary tumor radiotherapy (abscopal effect), especially in combination with immune-checkpoint inhibitors [25–30]. These data suggest that tumors at anatomically distant sites may influence one another’s growth dynamics through different biological processes briefly referenced above, and that true ‘local’ therapy may not exist. Thus, concomitant resistance could potentially be harnessed to maintain an environment of growth inhibition of post-treatment residual disease [16]. A thorough understanding of patient-specific burden limits, the systemic interplay dynamics of multiple tumor sites and their therapeutic modulation is needed.

Mathematical modeling has proven useful to describe tumor growth dynamics, and multiple highly integrated interdisciplinary efforts have identified and established various growth laws to describe and even predict how tumors change over time with and without different therapies [31–33]. To date, however, these works have rarely considered the interaction effects of multiple tumors within the same organism [34, 35]. There is a dire clinical need to identify patients with a high risk for metastases and to forecast their systemic disease behavior. Metastases and their symptoms are responsible for the overwhelming majority of cancer-related deaths [5]. However, mathematical modeling is also useful to explore these interaction effects in systemic cancerous diseases to understand which dynamics influence the systemic interconnectivity and how they can be altered to improve clinical outcomes. Mathematical modeling can help explore dynamic interactions and how to best harness them for clinical utility. A mathematical model of tumor and tumor-distant interactions has previously supported a systemic control of tumor growth through angiogenesis and proliferation inhibition, but lacks clinical applicability [35]. Notably, this model’s analysis rejected any inter-tumor competition. Here, we revisit the notion of systemic control of tumor growth through the novel concept of a systemically shared carrying capacity and validate it against the same data as well as new experimental data across multiple cell lines and mouse models.

### Materials and methods

**Fig. 1:**
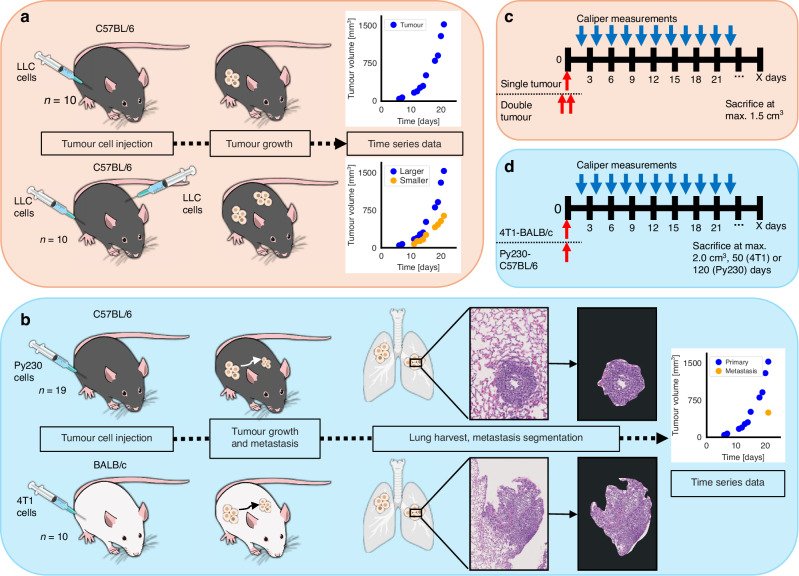
The setup and data extraction for the two experiments. **a** One or two injections of tumor cells are performed in two different groups of mice, and their sizes are followed over time with caliper measurements. **b** Two different cell lines are injected in two different mouse models. Primary tumor is again followed with caliper measurements. At sacrifice, the metastatic nodule size in the lungs is determined through H&E staining and image analysis. **c**, **d** The experiments’ designs. The underlying dynamics are examined through the concept of a shared systemic carrying capacity in two, respectively appropriate mechanistic mathematical models. Different model assumptions and restrictions are tested to identify the parsimonious model formulations (details in the text).

To study the systemic interconnectivity of primary and metastatic tumors, we examined different mathematical and experimental models. An overview of the experimental designs and data preprocessing is shown in Fig. 1.

### Mouse experiments

For the first experiment, previously published data of two groups of 10 male 6–8 weeks old C57BL/6 mice each were used [35]. The control single tumor group (ST group) was inoculated with a single subcutaneous injection of murine Lewis lung carcinoma (LLC) cells, and the second dual tumor group (DT group) was inoculated with two identical cell counts of LLC cells on opposite caudal halves of the back. Tumor size was measured daily with calipers up to a maximum of 1.5 cm^3^ when mice were sacrificed.

The second experiment was conducted using 2 different cell lines (aggressive and highly metastatic 4T1 and moderately aggressive Py230) in two different mouse strains (C57BL/6 and BALB/c) to evaluate the growth of primary tumors and corresponding metastases in the lung. BALB/c mice have a Th2 type immune response, whereas C57BL/6 have a Th1 type immune response [36]. Generally, the latter is considered more favorable in cancerous diseases [37]. We refer to these two groups as 4T1-BALB/c (10 mice) and Py230-C57BL/6 (19 mice).

### Animal ethical statement

All animal studies were performed in strict accordance with the NIH guidelines for the care and use of laboratory animals (Guide for the Care and Use of Laboratory Animals, 8th edition, National Research Council (US) Committee for the Update of the Guide for the Care and Use of Laboratory Animals, Washington (DC): National Academies Press (US); 2011). The experimental protocols were approved by the Institutional Animal Care and Use Committees (IACUC) of the Johns Hopkins University and the animals were treated according to the policies and guidelines of the Johns Hopkins University Animal Care and Use Committee, Baltimore, Maryland. Mice were fed normal diet and water *ad libitum*. All cages were maintained in the normal 12 h light/12 h dark cycle. All mice were monitored closely for any signs of distress or pain throughout the study period. No animal randomization or blinding was performed.

### Cell culture (Experiment 2)

Both 4T1 and Py230 cell lines were purchased from American Type Cell Culture (ATCC). 4T1 cells (ATCC CRL-3279, RRID CVCL_AQ08) were grown in RPMI media (Thermo Fisher Scientific, Waltham, MA) with 10% FBS (MilliporeSigma, Burlington, MA) and Py230 cells (ATCC CRL-2539, RRID CVCL_0125) were grown in F-12K media (Thermo Fisher Scientific, Waltham, MA) with 5% FBS and 0.1% MITO+ serum extender (Corning, NY). Cells were tested for mycoplasma routinely with Hoechst staining and confirmed negative. Both cell lines were authenticated using a short tandem repeat analysis (data provided in the repository) and matched against the ATCC database to ensure their genetic origin. Cell lines between 10 and 15 passages were used for mouse injections.

### Tumor cell injection (Experiment 2)

Female BALB/c or C57BL/6 mice (8 weeks old) were purchased from Jackson Laboratories (Bar Harbor, ME; RRIDs IMSR_JAX:000651 and IMSR_JAX:000664). All mice were allowed to acclimatize for 1 week prior to tumor cell injection. 4T1 cells (25,000 in 0.05 ml PBS) or Py230 cells (3 million in 1:1 Matrigel/PBS—0.05 ml) were orthotopically injected into the inguinal mammary fat pad of BALB/c (*n* = 10) or C57BL/6 (*n* = 11) mice. Tumor size was measured twice weekly with calipers to a maximum of 2.0 cm^3^ when mice were sacrificed. As additional endpoints, death or a maximum of 50 days after injection were chosen for 4T1-BALB/c and death or a maximum of 120 days after injection were chosen for Py230-C57BL/6. Upon sacrifice, lungs were collected from all mice, fixed in 10% formalin for 48 h, paraffin embedded and evaluated after H&E staining for metastatic nodules. For 4T1-BALB/c single slides were evaluated as all mice develop multiple nodules in lungs by the endpoint whereas for Py230-C57BL/6 four serial sections (100 µm apart) were stained with H&E for detecting tumor nodules. H&E slides were then digitized using NanoZoomer-SQ Digital slide scanner (Hamamatsu Photonics, Shizuoka, Japan; RRID SCR_023763) for further evaluation. Py230 control tumor and metastasis data from a published manuscript (*n* = 8) were also included to strengthen the analysis as only about 40% of these mice develop metastasis [38]. These cohorts were of same age and injected with cells exactly as described above.

### Estimation of metastasis sizes

To evaluate the sizes of the metastatic nodules on the H&E-stained slides, we developed in-house software to generate estimations of volumetric sizes of the metastatic nodules. In a first step, we segmented nodule regions in the tissues on the H&E slides with a deep learning image analysis algorithm [39]. For this, we utilized the published deep learning model architecture *SegFormer* [40] and trained it on published data sets [41, 42] to obtain annotations of active tumorous regions of interest on H&E stain slides of lung tissue. After this step and to address volumetric sizes of metastatic nodules that include necrotic regions, the images were post-processed in MATLAB R2024b (The MathWorks Inc., Natick, MA; RRID SCR_001622) with in-house implementations of dependencies from the Image Processing Toolbox. These created multiple two-dimensional areas that were combined to obtain a mask for annotations of metastatic areas on the original H&E slide. The identified nodules were confirmed by trained pathologists. From the post-processing and the two-dimensional areas, we derived three-dimensional volume estimates of the observed metastatic nodules assuming spherical shapes for all metastases. To estimate three-dimensional volumes of the metastatic nodules from two-dimensional annotated areas, we used the formula ${\mathrm{vol}}=\,{\frac{4}{3}r}^{3}\pi$ with the radius $r=\sqrt{\frac{{\mathrm{area}}}{\pi }}$ derived from a two-dimensional circle that had the corresponding area to the generated two-dimensional annotation.

### Two-tumor model: competition modeled with a shared carrying capacity

With intrinsic tumor growth rates *r*_1_, *r*_2_ and a shared carrying capacity *K*, the model for the connected Gompertzian tumor growth for each tumor *T*_1_ and *T*_2_ was formulated as

$$\frac{d{T}_{1}}{{dt}}={r}_{1}{T}_{1}\,{ln}\left(\frac{K}{{T}_{1}+{T}_{2}}\right){{\rm{and}}}\frac{d{T}_{2}}{{dt}}={r}_{2}{T}_{2}\,{ln}\left(\frac{K}{{T}_{1}+{T}_{2}}\right)$$

with the initial conditions ${T}_{1}\left(0\right)={T}_{{\mathrm{1,0}}}$ and ${T}_{2}\left(0\right)={T}_{{\mathrm{2,0}}}$. Without loss of generality, the larger tumor is denoted by *T*_1_ (*t*) at time *t*. This ensures that the DT group model is also applicable for the ST group with *T*_2_ (*t*) set to zero, i.e., ${T}_{{\mathrm{2,0}}}={T}_{2}(t)=0$. It then reduces to a one-tumor growth law with common Gompertzian growth. The model formulation with logistic growth dynamics is shown in Supplementary Materials.

### Metastatic tumor model with a shared carrying capacity

We extended this shared carrying capacity modeling approach (without increasing the number of model parameters) into a previously published mathematical model that consisted of a partial differential equation [43]. The size of a primary tumor at time *t* is denoted by ${x}_{{PT}}(t)$. Over time, the change of the tumor’s size $\frac{{{dx}}_{{PT}}}{{dt}}$ is defined through an ordinary differential equation with Gompertzian shape:

$${g}_{{PT}}\left({x}_{{PT}},\theta \right)=\,\frac{{{dx}}_{{PT}}}{{dt}}={a\,x}_{{PT}}\left(t\right){ln}\left(\frac{K}{\theta \left(t\right)}\right)$$

where *a* is the intrinsic growth rate of the primary tumor. This primary tumor can seed metastases. The change in time and in size of the metastases is defined through the partial differential equation

$$\frac{\partial \rho (x,t)}{\partial t}\,+\,\frac{\partial ({g}_{m}(x,\theta )\rho (x,t))}{\partial x}=0$$

where $\rho (x,t)$ denotes the density distribution of a metastasis of size *x* at time *t*. Further, the growth of a metastatic tumor is described by

$${g}_{m}\left(x,\theta \right)=\,\frac{{dx}}{{dt}}={a}_{m}\,x\left(t\right){ln}\left(\frac{K}{\theta \left(t\right)}\right)$$

with *a*_*m*_ the intrinsic growth rate of the metastasis of size *x*. The primary and metastatic tumors share a common systemic carrying capacity *K*, and their combined growth is mediated by the time-specific overall tumor load

$$\theta \left(t\right)=\,{x}_{{PT}}\left(t\right)+\,\int ^{\infty }_{1}\!\!\!\widetilde{x}\rho (\widetilde{x},t)d\widetilde{x}.$$

To formalize the seeding of new metastases, we choose the boundary condition

$$g\left(1,\theta \right)\rho \left(1,t\right)=\beta \left({x}_{{PT}},\theta \right)+\int ^{\infty }_{1}\!\!\!\!\beta \left(\widetilde{x},\theta \right)\rho \left(\widetilde{x},t\right)d\widetilde{x}$$

which introduces a new metastasis of cell size one depending on the current size of the primary tumor and the composition of the density distribution of the metastases at the corresponding time *t*.

Finally, the metastatic seeding function $\beta \left(x,t\right)$ is defined by the equation

$$\beta \left(x,\theta \right)=\mu \left(1-\frac{\theta (t)}{K}\right)\,{x}^{\chi }.$$

The fractal dimension parameter $\chi \in [{\mathrm{0,1}}]$ is a dimensionless unit that describes the propensity of colonies within a tumor able to form new metastatic colonies due to geometric and spatial constraints [44]. With this formulation, the entire tumor load is biologically feasible, and fewer metastases are seeded when the total tumor burden approaches the shared systemic carrying capacity. The initial assumptions are that there are no metastatic tumors ($\rho \left(x,0\right)=0\,\forall {x}$) and there is only the primary tumor featuring a specific mass of cells that have survived the inoculation and established as a tumor able to seed metastases, i.e., ${x}_{{PT}}\left(0\right)=\vartheta X(0)$. Without loss of generality, we assume that a tumor volume of 1 mm^3^ corresponds to 1 million cells [45]. Based on the in vivo experiment design, the value of *X*(0) corresponds to either 1 mm^3^ (experiment 1), 3 mm^3^ (experiment 2, Py230 cells) or 0.025 mm^3^ (experiment 2, 4T1 cells), derived from the inoculation. The parameter $\vartheta \in [{\mathrm{0,1}}]$ corresponds to the fraction of cells that successfully establish a primary tumor after inoculation. To determine whether a metastasis was clinically relevant, we assumed a threshold value of 3500 cells, which corresponds to about 0.24 mm in diameter for a spherical arrangement of the cells. Metastases below this threshold were considered as micro-metastases.

### Mathematical and statistical analysis

Numerical implementations and simulations of the different mathematical models, the model fitting routines, and the statistical analyses of the best fit results were implemented in MATLAB R2024b with the Global Optimization Toolbox. Model fits were performed with in-house code based on particle swarm optimization. The objective function to evaluate best fits for the first experiment was the sum of squared errors for fitting the data derived from the two-tumor group and the control group. The objective function for evaluation of the best fits from the second experiment was the weighted sum of relative errors of the primary tumor and volumes of metastases to account for different orders of magnitude between primary and metastatic lesions. For the optimization procedure, both objective functions were minimized with the *particleswarm* routine. Fits on the data from the second experiment were conducted in a nested optimization, i.e., different combinations of group-identical parameters were considered to reduce the number of free parameters. For all fitted parameter values we calculated relative standard errors as measures of parameter uncertainty. Parameter identifiability was assessed through profile likelihood curves, assuming normally distributed measurement errors. For the fits towards the first experiment, we further scatter-plot the residuals and reported the corresponding normalized root mean square error (NRMSE).

To examine statistical significance of determined model parameter distributions between the different groups, we employed different hypothesis tests. To evaluate whether the fitted parameter values of the different groups are from different distributions we used the Ansari-Bradley test and the two-sample Kolmogorov-Smirnov test [46]. To examine whether parameter values are normally distributed, we used the Anderson-Darling test. To determine if the fitted parameters are samples from continuous distributions with equal medians we used the Wilcoxon rank sum test. Distribution plots were generated with Python (RRID SCR_008394).

Different model assumptions and simplifications as well as population-based nested parameters or subject-specific parameters yield many different simulation combinations that were fitted against the respective available data. These combinations for the two-tumor model are a shared, individual, and equal, or individual and unequal carrying capacity *K*; an equal or unequal growth rate *r*; population-specific (*r*, *K*, or *r* and *K*) or individual parameters; and the growth equation for Gompertz or logistic growth. To identify the respective parsimonious model formulation, we select the minimal Bayesian Information Criterion (BIC) of all mentioned combinations [47, 48]. The combinations for the tumor-metastasis model determine whether the individual parameters are population- or subject-specific.

## Results

### Gompertzian growth law fits the data from the single tumor experiment

**Fig. 2:**
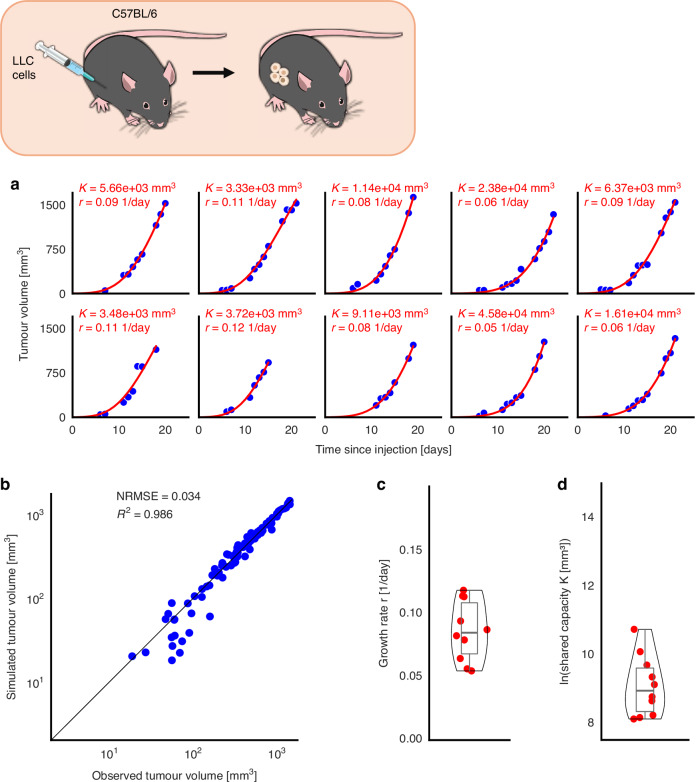
Model training to the 10 mice in the single tumor experiment. **a** Individual fits (curve) to experimental data (dots) of the single tumor. **b** Correlation plot and residuals between simulated and observed tumor volumes for all mice. **c** Distribution and individual parameters for growth rate *r*. **d** Distribution and individual parameters for carrying capacity *K*.

Analysis of the different considered growth laws and simulation combinations identified that Gompertz growth with an individual carrying capacity for the single tumor *T*_1_ (*t*) in the ST group provides the best fits among the tested models for the experimental tumor growth dynamics with an NRMSE of 0.034 (Fig. 2a, b). This is in line with the results reported in previous studies that identified Gompertz growth as the most likely underlying tumor growth model [31, 32]. Of note, the corresponding logistic growth fits for the ST group led to an NRMSE of 0.044 (Supplementary Fig. S3A). The median growth rate across all 10 mice was *r *= 0.085 day^−1^ (standard deviation 0.024, Fig. 2c) with a relative standard error of 3.08%, and the median carrying capacity was *K *= 7.74e3 mm^3^ (1.33e4, Fig. 2d) with a relative standard error of 4.46% (Table 1).

**Table 1:** The fitted parameter values and their distributions’ characteristics for the model corresponding to the first experiment, with equal intrinsic growth rate and shared carrying capacity for the primary and secondary tumors.

Parameter	Description	Unit	Median (Standard deviation) [DT]	Median (standard Deviation) [ST]	Relative standard error (%)
*r*	Intrinsic growth rate	day^−1^	0.085 (0.034)	0.085 (0.024)	3.08
*K*	Shared carrying capacity	mm^3^	1.22e4 (1.80e5)	7.74e3 (1.33e4)	4.46

### Two simultaneously growing contralateral tumors have equal intrinsic growth rates and share a systemic carrying capacity

**Fig. 3:**
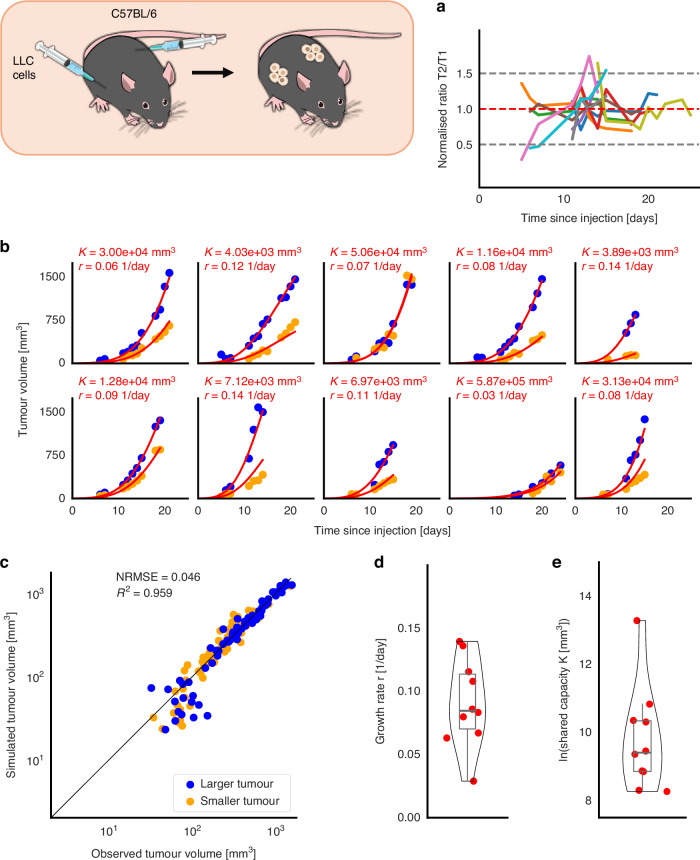
Model training to the 10 mice in the double tumor experiment. Blue: larger tumor, T1; orange: smaller tumor, T2**. a** Ratio of sizes of smaller vs. larger tumors over time divided by each respective mouse’s average ratio value. The gray dashed lines are the population’s corresponding Bland-Altman bands (±1.96 times the population’s standard deviation), and the red dashed line shows the normed ratio of one for reference. This plot indicates three outliers in the population. **b** Individual fits based on stated parameter values (red curves) to experimental data (dots). **c** Correlation plot and residuals between simulated and observed tumor volumes for all mice. **d** Distribution and individual parameters for growth rate *r*. **e** Distribution and individual parameters for carrying capacity *K*.

To achieve an identifiable model fit, it is imperative to know the initial number of cells that survive inoculation. Analysis of measurement variance suggests that the ratio between two simultaneously growing contralateral tumors in an individual mouse is constant over time, i.e., $\frac{{T}_{2}\left(t\right)}{{T}_{1}\left(t\right)}=\frac{{T}_{2}\left(0\right)}{{T}_{1}\left(0\right)}:=\varphi \,\forall t > 0$, or $\frac{{d}\left({T}_{2}{T}_{1}^{-1}\right)}{{dt}}=0$ for 7 of the 10 mice (70%) with an overall average ratio of 0.991 (standard deviation 0.231). The ratios relative to the respective mouse’s mean value are shown in Fig. 3a, revealing three outlier mice. We therefore assume mouse-specific constant ratios over time. This is independent of the choice of the net growth rate, rather, the assumption of constant ratios over time consequently leads to an identical ratio at *t *= 0 and equality of *r*_1_ and *r*_2_. This means that the initial tumor sizes were estimated from the ratios of the remaining longitudinal observations. The intrinsic growth rates were not assumed to be equal – the equality of the two rates is a consequence of the assumption that the ratios are constant over time. From this, we derive that the intrinsic growth rates of the two tumors are identical, ${r}_{1}={r}_{2}$, and we can consequently abstract the initial tumor volumes. Without loss of generality, ${T}_{2}(t)$ stands for the smaller tumor: ${T}_{{\mathrm{2,0}}}={\varphi {T}}_{{\mathrm{1,0}}}$ and $\varphi \in \left[{\mathrm{0,1}}\right]$.

The Gompertz model outperformed the logistic model, and the analogous analysis with logistic growth is shown in the Supplementary Fig. S1. The Gompertzian model with the smallest BIC (NRMSE = 0.046) featured an identical intrinsic growth rate with a median of *r *= 0.085 day^−1^ (0.034) for the two tumors, a shared carrying capacity with a median of *K *= 1.22e4 mm^3^ (1.80e5), and the initial condition of 1mm^3^ ≈ 10^6^ cells for the larger tumor (Fig. 3b, c and Table 1). The corresponding logistic growth model led to an NRMSE of 0.053 (Supplementary Fig. S3B). With the assumption of an equal intrinsic growth rate for both tumors, we receive that the effective net growth (i.e., the absolute rate of volume increase) of the smaller tumor is always smaller than the one of the larger tumor. Since $\frac{d{T}_{1}}{{dt}}=r{T}_{1}\left(t\right)\,{\mathrm{ln}}\left(\frac{K}{{T}_{1}\left(t\right)+{T}_{2}\left(t\right)}\right)$ and $\frac{d{T}_{2}}{{dt}}=r{T}_{2}\left(t\right)\,{\mathrm{ln}}\left(\frac{K}{{T}_{1}\left(t\right)+{T}_{2}\left(t\right)}\right)$, and ${T}_{1}\left(t\right) > {T}_{2}(t)$, it follows that $\frac{d{T}_{1}}{{dt}} > \,\frac{d{T}_{2}}{{dt}}$. This means that the initial number of cells that survive the injection at both injection sites contribute to the later differences in tumor volumes. This effect weakens when the ratio between the two tumors *φ* is closer to one, i.e., the closer the two tumor sizes are to each other. If ${T}_{1}\left(t\right)={T}_{2}\left(t\right)$ and ${r}_{1}={r}_{2}$, then the growth speed of both tumors is identical, i.e., $\frac{d{T}_{1}}{{dt}}=\frac{d{T}_{2}}{{dt}}$. Consistent with this reduction, profile likelihoods showed that the common growth rate *r* is well constrained and that the shared carrying capacity *K* is identifiable with finite 95% bounds (Supplementary Fig. S6A). This pattern reflects that the measured data do not approximate this saturation well, yet the curvature of the profiles excludes large deviations from the fitted value.

The distribution and medians of intrinsic growth rates *r* were not different for the single tumor and double tumor experiments (*p* = 0.82 [Ansari–Bradley], *p* = 0.97 [Kolmogorov–Smirnov], *p* = 0.77 [Wilcoxon rank sum]). Similarly, the distribution and medians of the shared carrying capacities *K* were not different for the single tumor and double tumor experiments (*p* = 0.1 [Ansari–Bradley], *p* = 0.68 [Kolmogorov–Smirnov], *p* = 0.11 [Wilcoxon rank sum]). However, the Anderson-Darling test rejected the null hypothesis that the fitted carrying capacities from both groups were samples from a normal distribution (*p* < 0.0005 [double tumor] and *p* < 0.01 [single tumor]). The parameter distributions are shown in Fig. 3d, e.

### Shared systemic carrying capacity, fractal dimension, and metastatic growth rates are mouse-specific

**Fig. 4:**
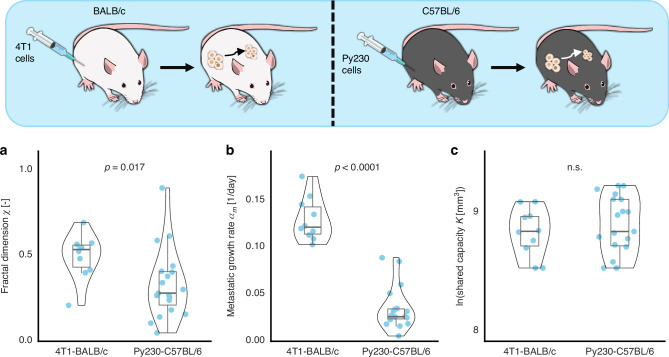
Analysis of fitted parameters for the second experiment reveals group-specific parameter distributions. Parameter distributions for the fractal dimension *χ* (**a**), the metastatic growth rate *a*_*m*_ (**b**) and the shared carrying capacity *K* (**C**) for the groups 4T1-BALB/c (left boxviolinplot in each panel, *n* = 10) and Py230-C57BL/6 (right boxviolinplot, *n* = 19). Statistical test: Wilcoxon rank-sum test.

**Fig. 5:**
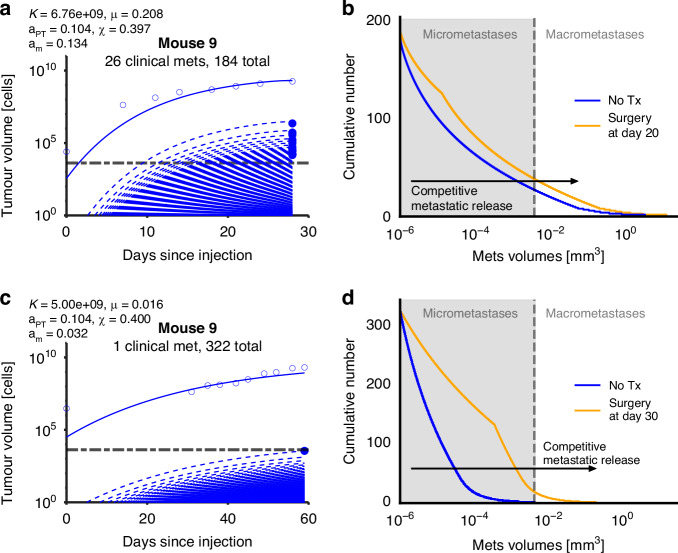
Example fits for one mouse from each group: in silico experiments of primary tumor removal identify competitive metastatic release. An example of the fits for a mouse from the 4T1-BALB/c group (**a**) and a mouse of the group Py230-C57BL/6 (**c**). Shown are the primary tumor measurements (blue circles) and simulations (blue solid curves) together with the measured volumes of the metastases (blue dots) and corresponding simulated metastases over time (blue dashed curves). The black dashed lines show the assumed detectability threshold of 3500 cells for a metastasis, corresponding to a diameter of 0.24 mm. We simulated an in silico experimental scenario of full surgical removal of the primary tumor for the mice presented in (**a**, **b**) at days 20 and 35, respectively. We identified massive acceleration of the metastatic growth after surgery (**b**, **d**). In particular, for the latter, a previously controlled and hardly discoverable single metastasis along with further micro metastases experience drastic competitive release and may manifest as metastatic disease. The plots show the cumulative size distribution of the metastases at the final day of the experiment (28 and 59 days, respectively) in the untreated (blue curve) and the treated setting (orange curve). The black arrows indicate the induced shift on the distributions.

We again used the BIC as a metric to identify the parsimonious model formulation for the tumor-metastases model. The BICs for the possible combinations are shown in the Supplementary. With this choice, the lowest BIC was achieved for the model reduction that introduces the parameters for intrinsic growth of the primary tumor *a*_*PT*_, the per cell per time rate of a tumor cell to form new metastatic cells *μ*, and the initial cell survival after inoculation $\vartheta$ as group-specific parameters. The three remaining parameters, namely the intrinsic growth rate of the metastases *a*_*m*_, the fractal dimension *χ* and the shared carrying capacity *K*, were identified to be subject-specific. The distributions of the three subject-specific parameters are shown in Fig. 4a–c. An example fit of a mouse of the 4T1-BALB/c group towards the experimental data is shown in Fig. 5a. The experimental measurements show volumetric tumor sizes at multiple time points, and sizes of metastases at endpoint. We show the best fit of the parsimonious model with shared carrying capacity for all tumors, that can describe the size of the primary and secondary tumors over time. All other individual best model fits for both groups of this model formulation are shown in the Supplementary. Parameter profile likelihoods in the metastatic model (Supplementary Fig. S6B, C) showed strong curvature for the primary and metastatic growth rates and the fractal dimension *χ*, while the shared capacity *K* and the initial cell survival after inoculation *ϑ* exhibit one-sided 95% bounds in particularly the Py230-C57BL/6 group.

### Model-trained parameters identify metastatic potential of cell line and mouse model

We next compared the model trained parameters between the 4T1-BALB/c and the Py230-C57BL/6 groups. As expected, the 4T1-BALB/c group had a higher median intrinsic growth rate of the primary tumor (*a*_PT_ = 0.104 vs. *a*_PT_ = 0.032; relative standard error 2.72e-5%), a higher median per cell rate of a tumor cell to form new metastatic cells (*μ *= 0.208 vs *μ *= 0.016 relative standard error 7.81e-4%), and a higher median metastatic growth rate (*a*_m_ = 0.120 vs. *a*_m_ = 0.026; relative standard error 2.58e-5%, *p* < 0.0001 [Wilcoxon rank sum]). Also, the parameter *χ* that describes the propensity of cells within a tumor able to form new metastatic colonies due to geometric and spatial constraints was significantly higher in the 4T1-BALB/c group compared to the Py230-C57BL/6 group (*χ *= 0.531 vs. *χ *= 0.279, *p* = 0.017 [Wilcoxon rank sum]) (Table 2). These relations between the population-specific parameters of the two different groups are in line with the biology-based expectations of 4T1 being aggressive and highly metastatic, and Py230 being moderately aggressive. Of interest are the almost identical median carrying capacities of *K *= 6.81e3 and *K *= 6.80e3 (*p* = 0.46 [Wilcoxon rank sum]) and fraction of cells surviving the initial inoculation. (*ϑ *= 0.014 vs. *ϑ *= 0.011; relative standard error 3.30e-5%). Perturbations within the 95% profile bounds preserved the qualitative change in the number of total and clinically relevant metastases (Supplementary Figs. S7 and S8).

**Table 2:** The fitted parameter values and their distributions’ characteristics for the model corresponding to the second experiment, with shared carrying capacity for the primary and metastatic tumors.

Parameter	Description	Unit	Median (standard deviation) [4T1-BALB/c]	Median (standard deviation) [Py230-C57BL/6]	Relative standard error (%)
*a*_*PT*_	Intrinsic growth rate of the primary tumor	day^−1^	0.104 (−)	0.032 (−)	2.72e-5
*a*_*m*_	Intrinsic growth rate of the metastases	day^−1^	0.120 (0.129)	0.026 (0.200)	2.58e-5
*K*	Shared carrying capacity	mm^3^	6.81e3 (1.35e3)	6.80e3 (1.77e3)	8.80e-4
*μ*	Per cell rate of a tumor cell to form new metastatic cells	day^−1^ cell^−1^	0.208 (−)	0.016 (−)	7.81e-4
*χ*	Fractal dimension: fraction of the tumor’s cells that may metastasize	-	0.531 (0.023)	0.279 (0.022)	3.09e-5
*ϑ*	Fraction of cells surviving the initial inoculation	-	0.014 (−)	0.011 (−)	3.30e-5

### Shared carrying capacity model simulates competitive release of metastases after primary tumor surgery

We next used the developed and calibrated model to simulate counterfactual experiments. Simulated removal of the primary tumor at day 20 for a mouse of the 4T1-BALB/c group indicates competitive release of previously suppressed metastases and acceleration of the effective seeding and growth of the metastases. Without surgery, the mouse had 16 detected metastases at the experimental endpoint (Fig. 5a), and 26 simulated clinically relevant metastases as per our uniform threshold value. After simulated surgery, the concomitant suppression from the primary tumor is released yielding 36 (+38%) clinically relevant metastases at simulated endpoint along with growth acceleration of all clinically relevant metastases (Fig. 5b). In a counterfactual simulation of inhibited metastatic seeding, the primary tumor grows faster and to a larger value, indicating bi-directional concomitant systemic interconnectivity (Supplementary Fig. S2A).

### Model-trained parameters identify conditions for metastasis control after primary tumor surgery

A more heterogeneous response was found in the counterfactual simulations of the Py230-C57BL/6 group. A mouse that had a single clinically relevant metastasis at experimental endpoint showed competitive release of metastatic disease to 18 clinically relevant metastases after simulated surgery (Fig. 5c, d). However, simulation of surgical removal of the primary tumor of another mouse that did not have any evidence of metastatic disease at experimental endpoint did not exhibit a systemic release of metastases after surgery as the full tumor burden had been excised (Supplementary Fig. S2B). These mice featured different mouse-specific parameters. Mouse 9 had a metastasis growth rate of *a*_m_ = 0.032 day^−1^, a fractal dimension of *χ *= 0.400 and a shared carrying capacity of *K *= 5.00 cm^3^; mouse 15 had a metastasis growth rate of *a*_m_ = 0.034 day^−1^, a fractal dimension of *χ *= 0.156 and a shared carrying capacity of *K *= 9.31 cm^3^. To evaluate the influence of the value of the shared carrying capacity parameter in these two simulations, we repeated the surgery-free simulations using the shared carrying capacity value of the respective other mouse. The simulations did not differ greatly, and we concluded that it is not the shared carrying capacity value, but rather the metastatic growth rate and the cell propensity for metastasis (fractal dimension), that drive the presence and aggressiveness of metastases (Supplementary Figs. S5, S9, and S10). This suggests that successful control after surgery is determined by the strength of the competitive release, which is regulated by the parameters *a*_*m*_ and *χ*.

## Discussion

We presented a novel mathematical modeling approach that simulates the dynamics of multiple concurrently growing tumors. Testing multiple models on different cancer cell lines and mouse models identified a parsimonious Gompertz growth model with a shared carrying capacity. This simulates systemic, bi-directional interconnectivity of tumors within the same host to study therapeutic perturbation of these coupled dynamics. This model demonstrated that the effective tumor net growth (i.e., the absolute rate of volume increase) of the larger tumor is always larger than the smaller tumor, and that their difference is defined by the ratio between the two tumor sizes. This is due to, at least in part, the mathematical formulation, but also in line with previous investigations and observations [35, 49, 50]. Removing the primary tumor’s suppressive influence by treating the largest tumor may create novel stimuli for smaller tumors [51, 52] and ignite acceleration of metastatic seeding and metastatic growth through competitive release from concomitant competition. This is supported by the profile likelihoods that indicate the key drivers of our counterfactual results are practically identifiable with finite 95% bounds, and that parameters with flatter profiles show one-sided bounds but do not tilt the qualitative behavior in uncertainty.

To our knowledge, this is the first mathematical model with a shared carrying capacity for primary and metastatic tumors that has been calibrated on multiple cancer cell lines and mouse models to compare their quantitative differences. Through nested optimization and use of information criteria, we were able to reduce the number of subject-specific parameters and identified three of the six used parameters to be defined by the group that the respective mouse belonged to. Since the primary and metastatic tumors are interconnected, we were able to identify the parameters from a single measurement time point for the distribution of the metastases. The three subject-specific parameters are the intrinsic metastatic growth rate, the fractal dimension, and the shared systemic carrying capacity. This aligns with previous modeling work, which found that similar parameters can serve as predictive computational biomarkers in a model formulation with non-shared capacity [53]. Even though biological deceleration with size motivated Gompertz growth a priori, we performed the entirely analogous analysis with a coupled logistic growth model and checked the exponential growth curve fits, which showed less agreement with the experimental data than the logistic or Gompertz fits, especially during early time measurements (NRMSE = 0.168 and 0.117, respectively; Supplementary Figs. S3 and S4).

The differences we found between the two groups in these parameters are consistent with their known biological differences of mouse models and cell lines, which were intentionally chosen to describe different degrees of tumor interactions and metastatic disease. Further, we found a significant difference between the two groups in the metastatic growth rate and the fractal dimension that describes the propensity of cells within a tumor able to form new metastatic colonies due to geometric and spatial constraints. The other subject-specific parameter, the systemic carrying capacity, was not found to feature statistically significant difference in distribution between the two groups, which is in line with the observations from the two-tumor model and the first experiment. Previous reports show evidence that the carrying capacity for single tumors is essentially species-specific, but with a considerable range of degrees of subject-specific restraint [1, 54, 55].

The shared carrying capacity reflects a global host constraint such as total vascular supply, nutrient availability or systemic immune tolerance, rather than local tumor microenvironmental properties. The shared carrying capacity parameter was remarkably comparable between the two groups and suggests that the ultimate tumor burden for an individual mouse is determined through host-level resources. Local microenvironmental factors clearly modulate individual lesion growth, but this finding across two biologically different cohorts implies a dominant, systemic limitation. This conceptually aligns with concomitant resistance, where the presence of one lesion suppresses the outgrowth of other lesions. In our formulation, an increase in the total burden *θ*(*t*) reduces the effective growth term ln (*K*/*θ*), constraining all other lesions jointly. The metastatic disease is likely characterized by the parameters *a*_*m*_ and *χ*, where the former was identified as the intrinsic growth rate of metastases. The latter represents the metastatic propensity, which is governed by local microenvironmental factors, as mentioned above. We therefore propose that the shared capacity captures a physiological maximum that may be subject to context-dependent variation (in particular intrinsic cell proliferation properties, local microenvironment setups, the patient’s immune system, systemic inflammation and other factors contributing to the physiological state) and is not only the sum of local tissue-specific effects. Future studies should explore how organ-specific niches or interspecies differences may define and affect this systemic carrying capacity and the local contributing factors. The presented model and simulation results motivate experiments to identify the biological correlates of the shared carrying capacity, the intrinsic metastatic proliferation rate and the metastatic propensity, for instance, total vascular capacity, local angiogenesis or endocrine factors, and to test in silico whether modulating these can alter the overall tumor burden. In particular, favorable shifts may be relevant for the identification of effective treatments for systemic cancer disease, whereas unfavorable ones may indicate treatment failure even before its application.

The model provided a novel, mechanisms-agnostic insight into concomitant resistance and how systemic interconnectivity between multiple tumors in the same host can potentially be altered. In agreement with current scientific literature, our analysis demonstrated that metastatic growth could accelerate after surgery. This has been widely attributed to the increased expression of pro-inflammatory and angiogenic signaling molecules and cytokines after surgery [56–61], and may result in metastatic outgrowth peaking at specific times after surgery [20, 62–64]. Other data demonstrated additional mechanisms, including decreased access to nutrients with inhibition of angiogenesis [65], T cell-triggered immune responses that alter the metastatic tumors’ microenvironment [66, 67], and serum factors that inhibit proliferation independent of the immune system [66]. Of interest, systemic inflammation has been identified to accelerate metastatic growth [68], and primary tumors with an immunosuppressive microenvironment can lead to non-responding metastases [69]. On the other hand, local radiation has been demonstrated to trigger a broad variety of effects on distant metastases, ranging from competitive release to an abscopal effect, especially when combined with immunotherapy [25, 28–30]. A recently formulated hypothesis of cancer hormesis adds the theoretical idea to implant artificial benign tumors as competitors against more aggressive metastatic tumors [70].

The parsimonious model formulation is optimal among the tested contexts and is, of course, an approximation and simplification of the vast number of underlying pathways and dynamics. We assumed constant size ratios for the double tumor experiment which consequently identified equal growth rates. These important approximations and assumptions therefore form central limitations and a contextualization of our approach. While the presented model formulates the competitive interactions among tumors through a shared carrying capacity, it does not explicitly account for cooperative mechanisms, such as immunosuppressive signaling or tumor-induced angiogenesis, that clearly may influence tumor dynamics [71–74]. We modeled the effects of surgery in silico, and the simulations reproduced phenomena reported in the literature, but experimental confirmation of these effects is pending. Calibrating this approach to data from experiments comparing metastatic distributions with and without surgical removal would strengthen its validation. In particular, forecasting metastatic growth after surgery and validation in prospective experiments may verify its trustworthiness, even though observing metastatic growth below clinically detectable thresholds in vivo is challenging. Potential experiment design includes randomization of mice into primary tumor resection vs. no resection at predefined sizes, and to longitudinally track metastases for quantification of their distribution at matched endpoints to prospectively validate post-surgical disease dynamics. Additionally, we have assumed a constant carrying capacity throughout this work. Seminal studies by Hahnfeldt and colleagues demonstrated a putative dynamic carrying capacity that is altered as the tumor grows and evolves [75]. Similarly, a combined body of work by Zahid and Enderling and colleagues discussed treatment effects on the carrying capacity [76, 77]. These nonlinearities will further increase complexity and will require additional data and future work to test and validate. Appropriate experiment design may focus on pharmacologically modulated seeding and the collection of systemic biomarkers such as angiogenic or inflammatory panels to explore correlates of the shared carrying capacity. In this work, we deliberately developed a parsimonious model formulation to capture the key dynamics without overfitting to secondary mechanisms, and to build upon in the future. We explicitly did not focus on a predetermined mechanism. On the one hand, this motivates further research into identifying the putative mechanisms but also suggests that the proposed model may serve as a formulation to summarize these mechanisms in a parsimonious formulation. This demonstrates that tumors at anatomically distant sites are interconnected.

While the focus of this work was on mouse data, we expect that extensions and scaling of this approach from mouse to human including individual cancer types and different treatment applications are possible, and that they can serve as tools to evaluate response and outcomes to counterfactual treatments within a digital twin framework [78–80]. Future work should also aim to estimate patient-specific model parameters from limited pre-clinical and clinical longitudinal data. This enables us to derive a risk score to determine whether metastatic lesions have likely formed and are below level of detection, or whether a primary tumor truly represents localized disease. Such an approach aligns with the notion that in the presence of secondary tumors, particularly micro-metastases, truly local therapy may not exist [81].

## Supplementary information

Supplementary Material

## Data Availability

Data corresponding to the first animal experiment is already published [35]. Data from the second experiment is available through the GitHub repository https://github.com/psch-mdacc/DataSysCarrCap and the Johns Hopkins Research Data Repository 10.7281/T1/KTOG9W. Code is available through the GitHub repository.
