# Supplementary material for: Gompertz growth with a shared carrying capacity optimally simulates primary and metastatic tumor growth dynamics
Source: Br J Cancer. 2026 Feb 24;134(8):1138–49. doi: 10.1038/s41416-025-03306-9 (PMC13035863; doi:10.1038/s41416-025-03306-9)
Supplement: Supplementary file 1 — Supplementary Material [file 41416_2025_3306_MOESM1_ESM.docx]

## Supplementary

**Bayesian Information Criterion (BIC) calculation**

With $p$ the number of free parameters, $n$ the number of data points and $SSE$ the sum of squared errors of the model fit to the data, we receive the $BIC$ as

$$BIC=p*\ln\left( n \right)+n*\ln\left( \frac{\sum SSE}{n} \right)$$

The $BIC$is a standard model selection criterion and a metric to quantify the tradeoff of parameter reduction and the goodness-of-fit to penalize model complexity under consideration of the goodness-of-fit. The model that features the minimum $BIC$ of some set of reference models is selected as the most parsimonious model (1,2).

1. Konishi S, Kitagawa G. Information Criteria and Statistical Modeling. Springer New York, NY; 2008.

2. Zhang J, Yang Y, Ding J. Information criteria for model selection. WIREs Computational Statistics **2023**;15


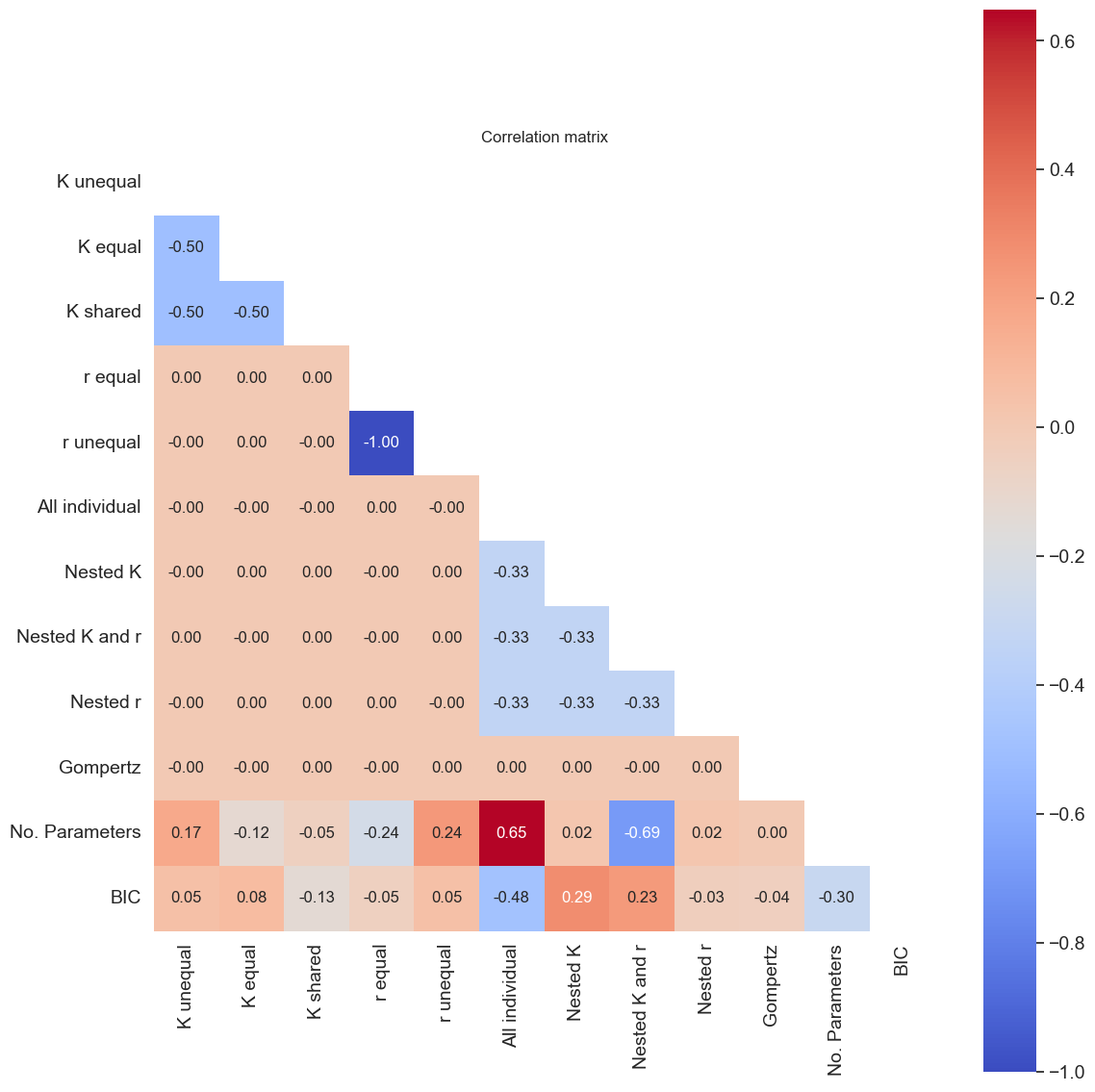

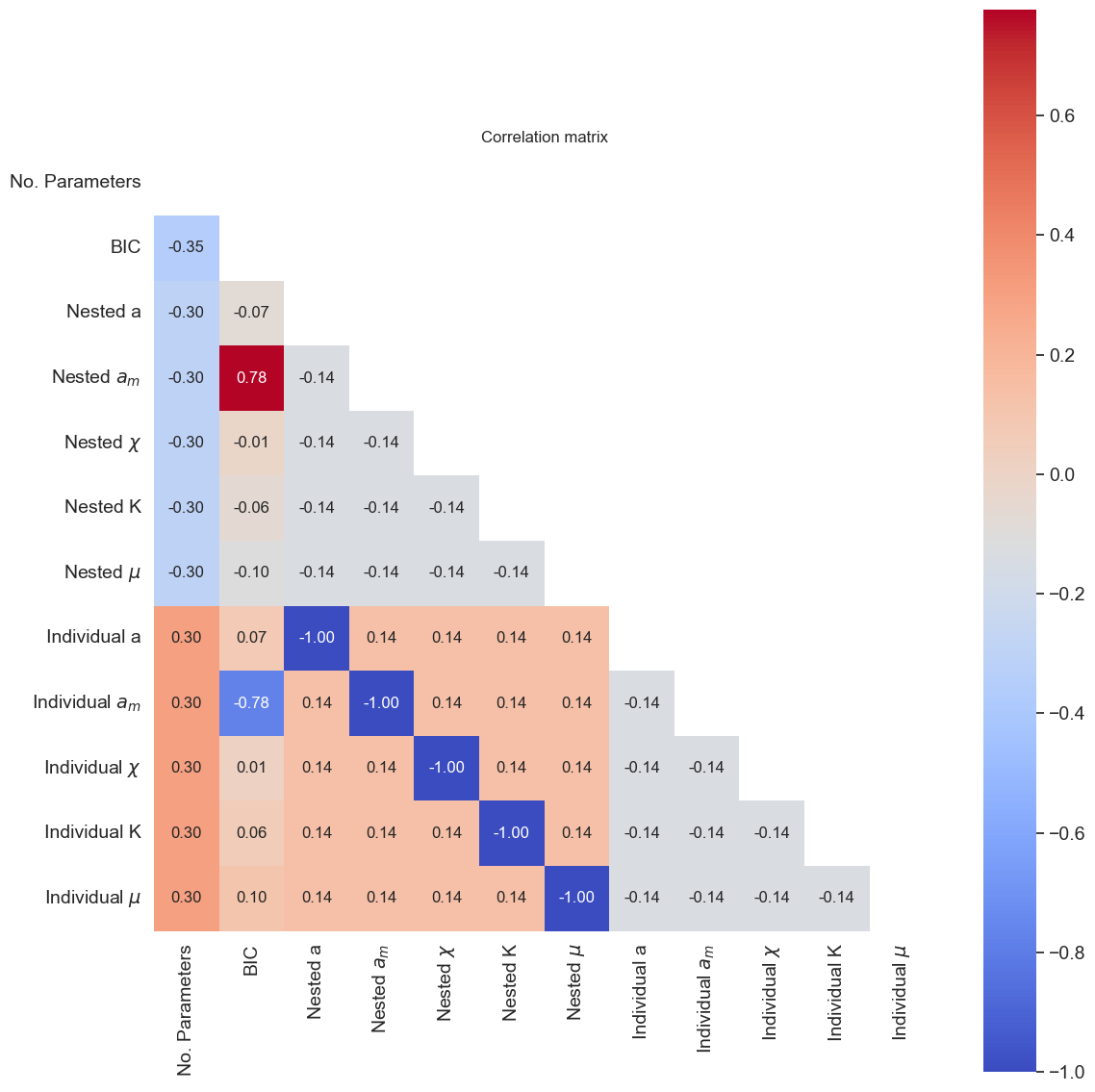

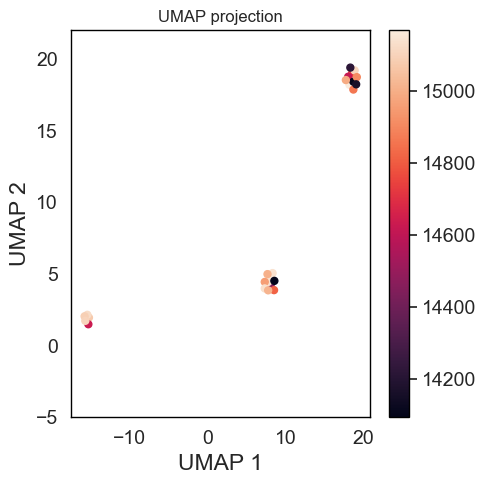

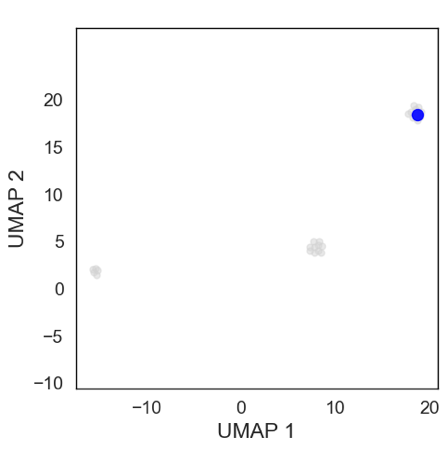

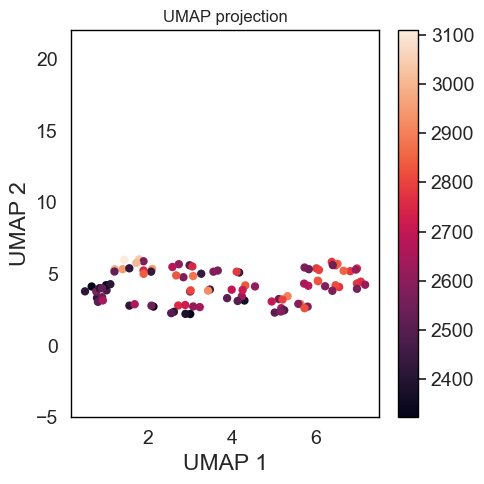

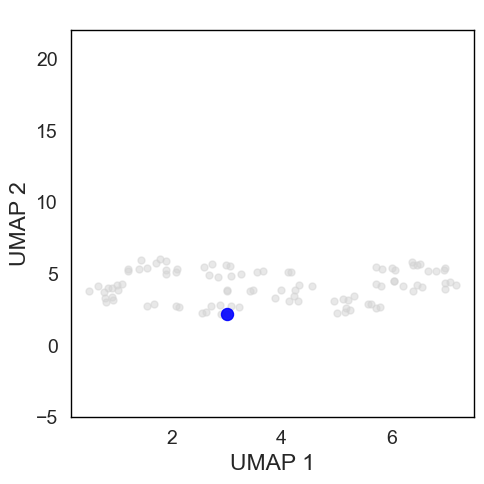


**B**

**Figure S1. The evaluations for the parsimonious model selections to analyze the compositions of their individual settings.** For the two-tumor model **(A)** and the tumor-metastasis model **(B)**: a lower value corresponds to a lower BIC when the feature is present. For instance, the model combinations for the two-tumor model have a strong linear correlation of 0.65 between the increasing number of free parameters and the state that all models have fully individual (and no population-specific) parameters. Overall, this indicates the selection for the shared carrying capacity, and individual parameters (two-tumor) as well as the individual metastasis growth rate (tumor-metastasis). The plots to the right show the UMAP projection of the different model combinations, and the middle plots highlight the position of the optimal model showing the minimal BIC value.

**A**

**Plots for the parsimonious model formulation for metastatic disease**

The following plots show the simulations of the individual subjects and their respective data with the parameter values determined from population-based nested fitting.

4T1 group


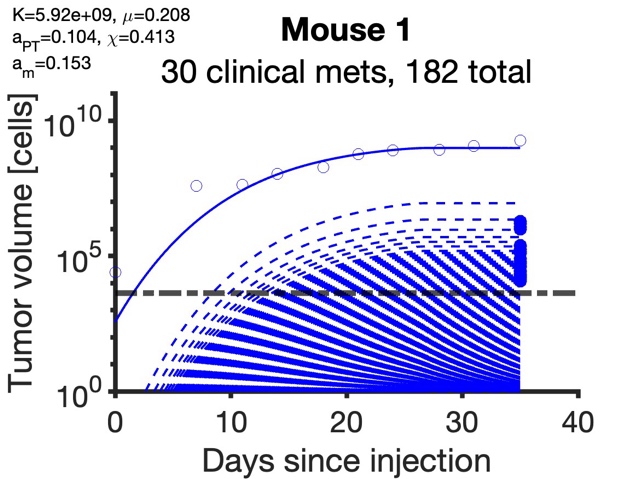

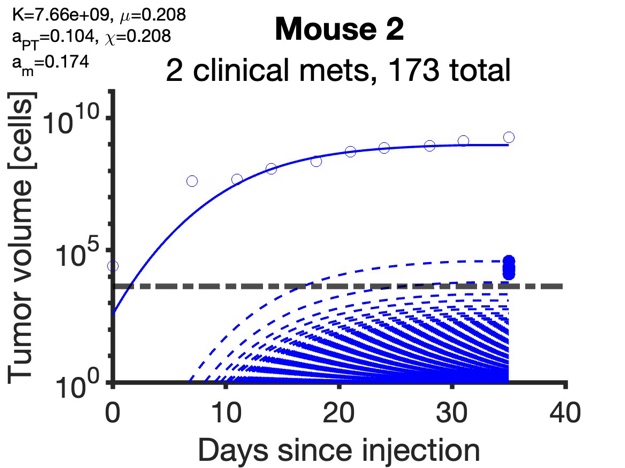


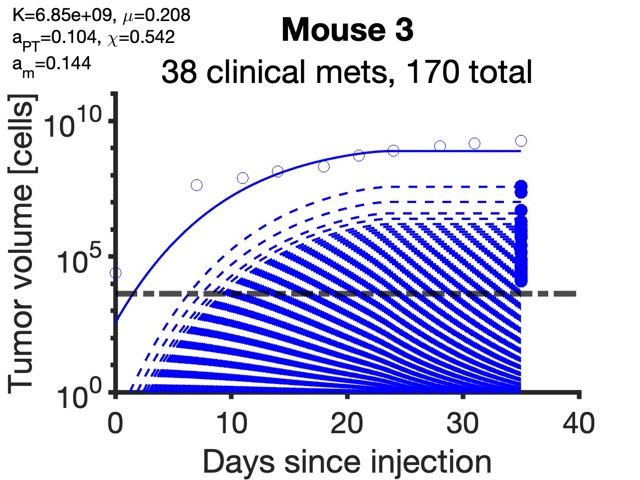

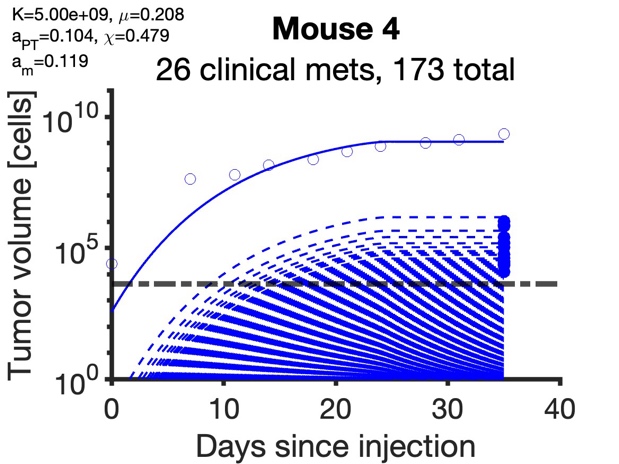


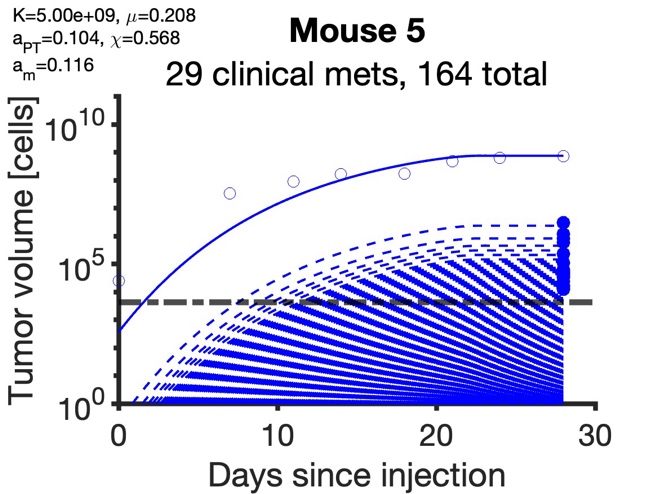

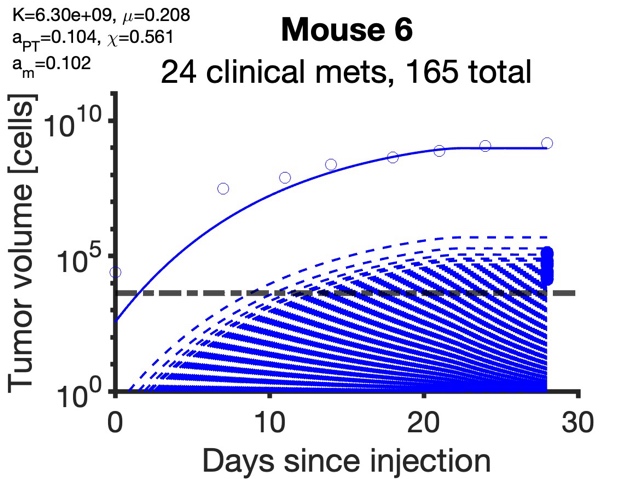


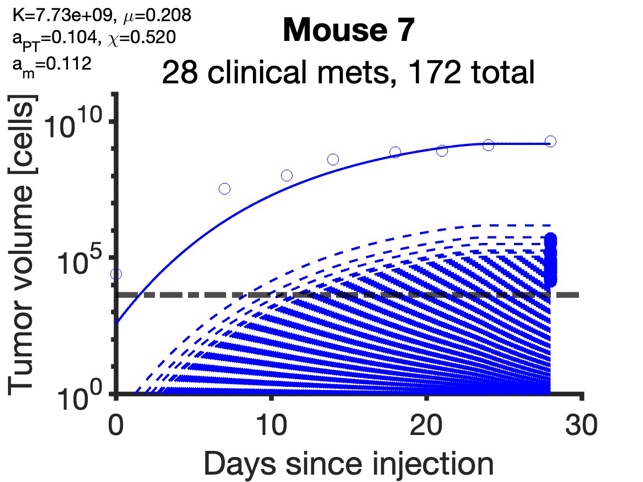

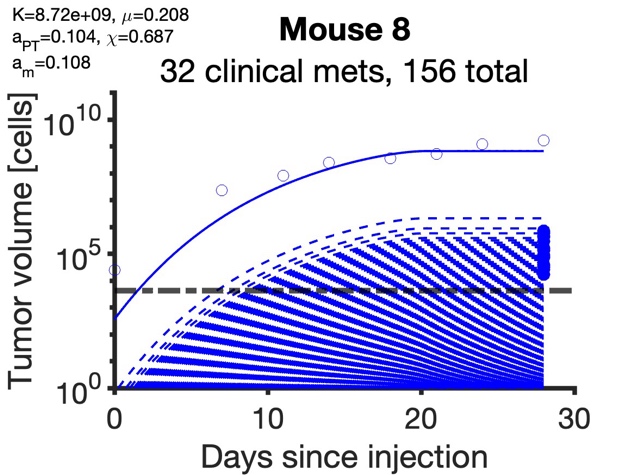


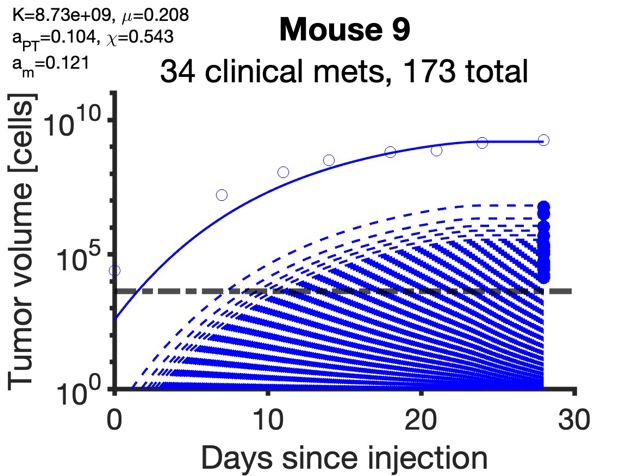

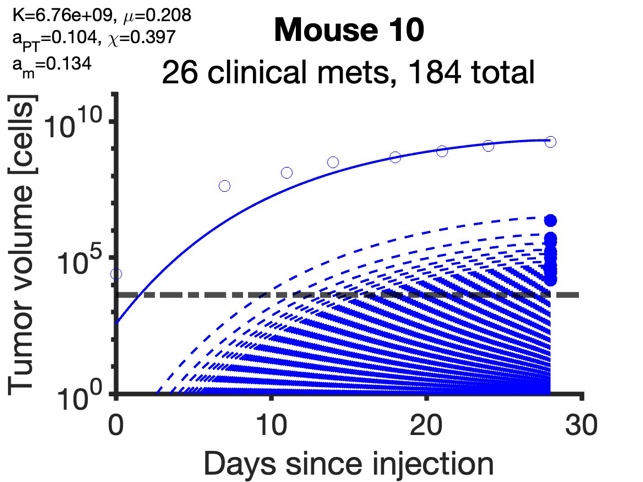


Py230 group


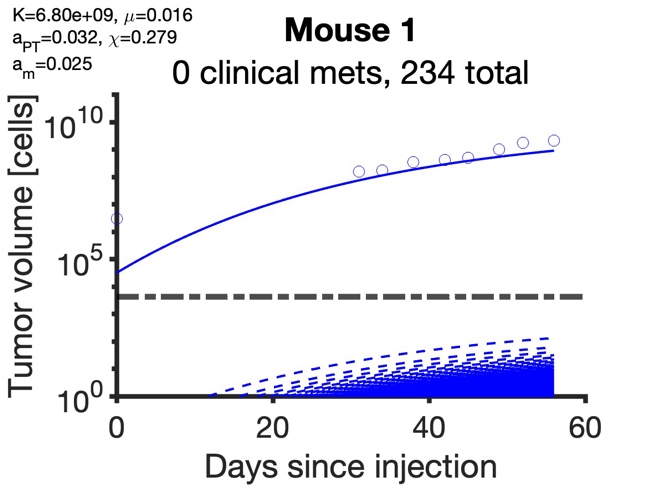

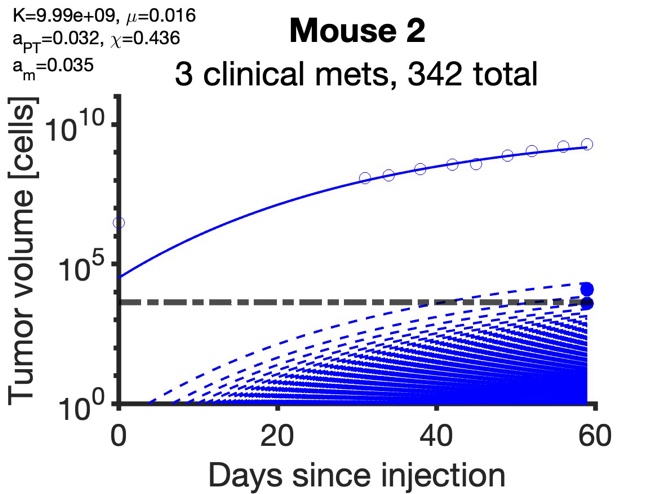


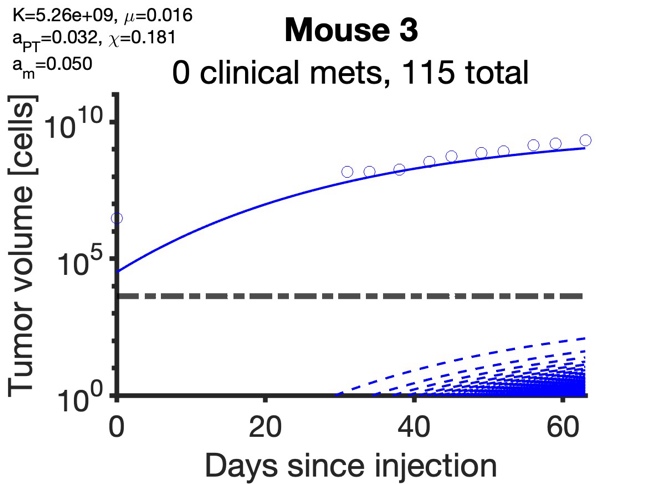

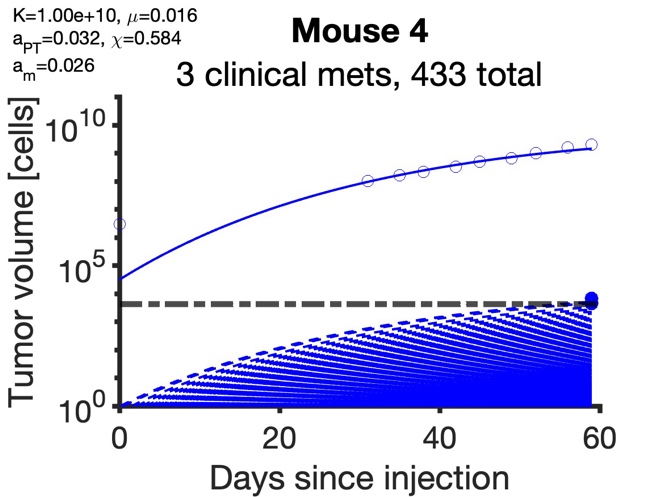


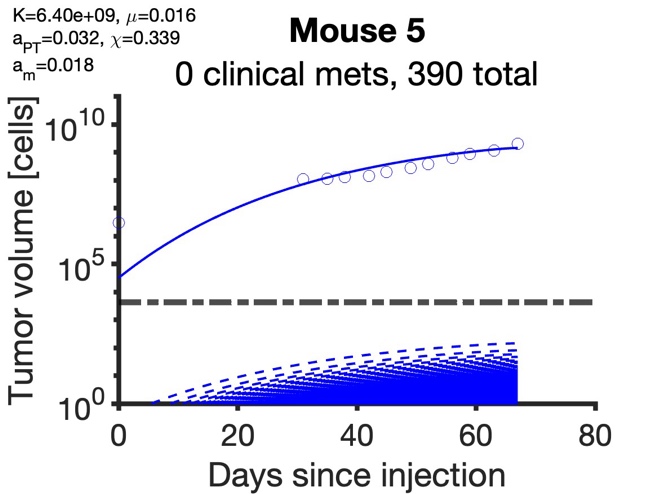

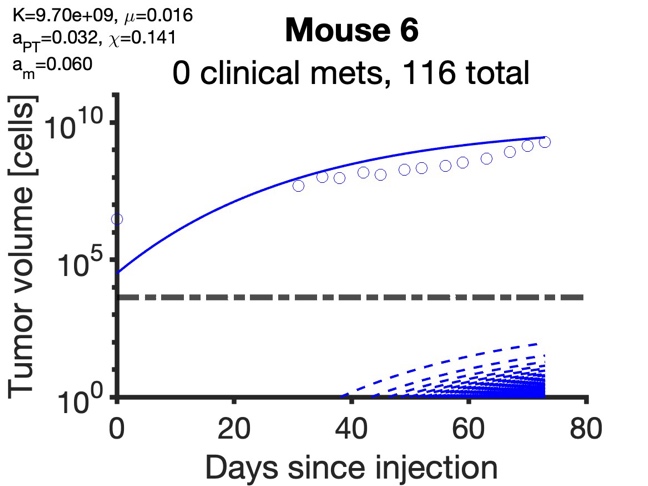


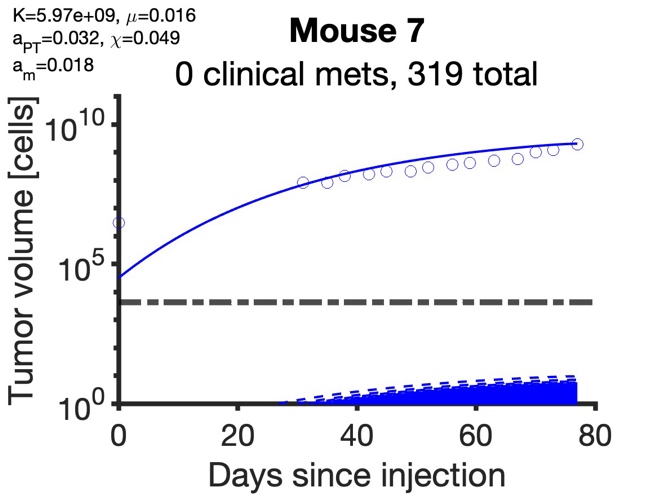

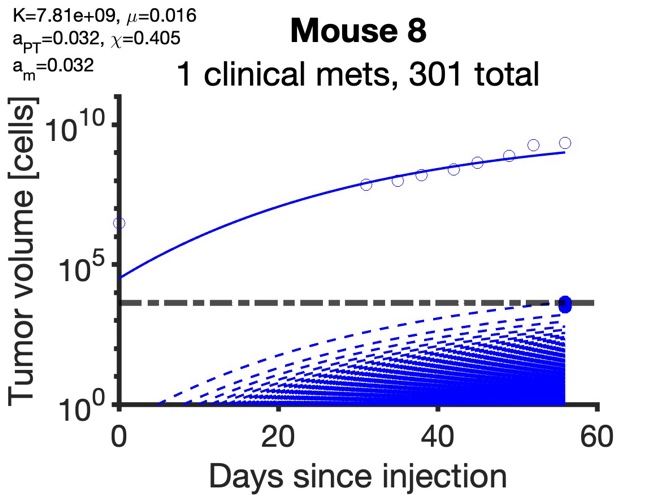


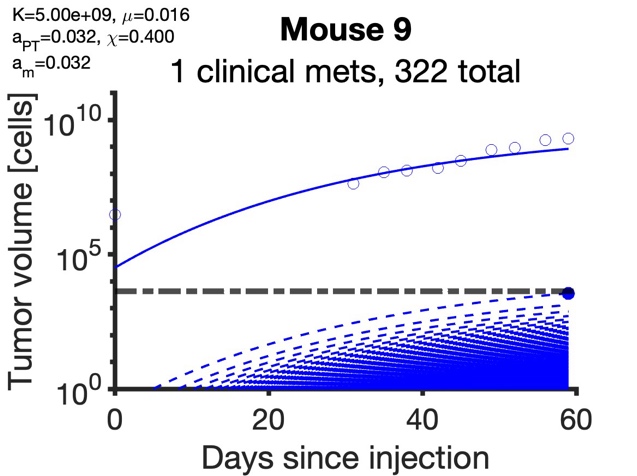

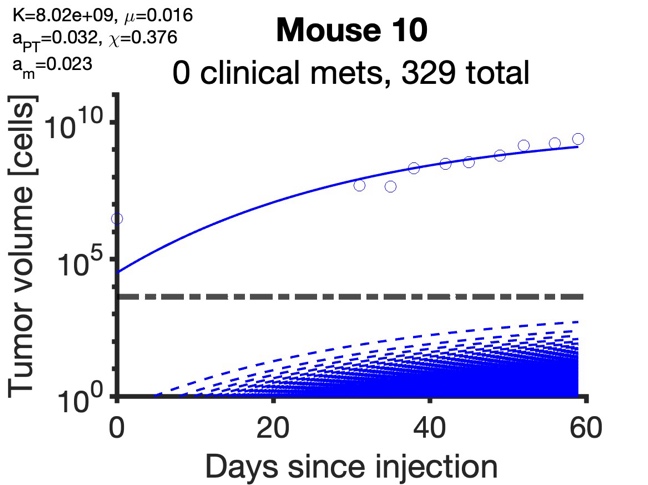


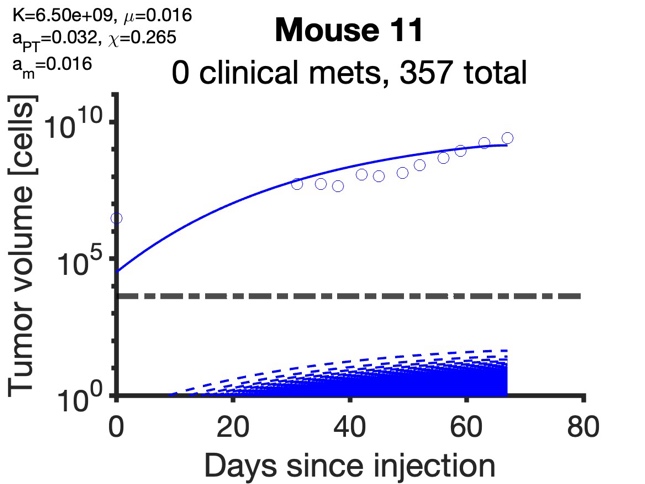

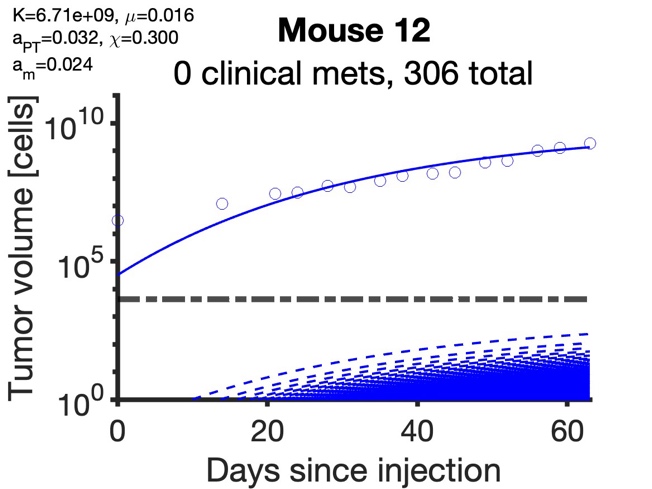


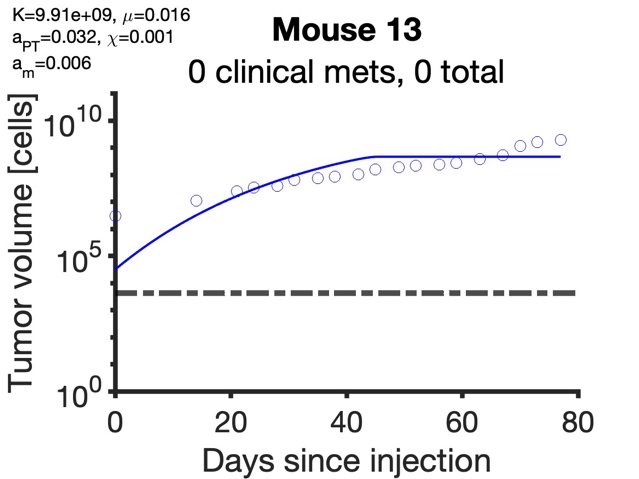

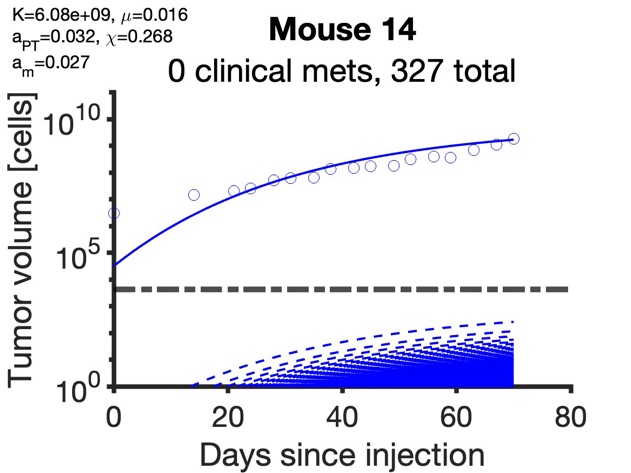


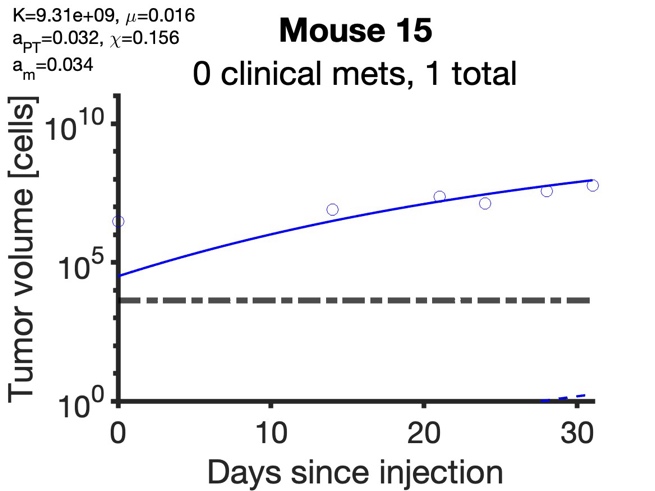

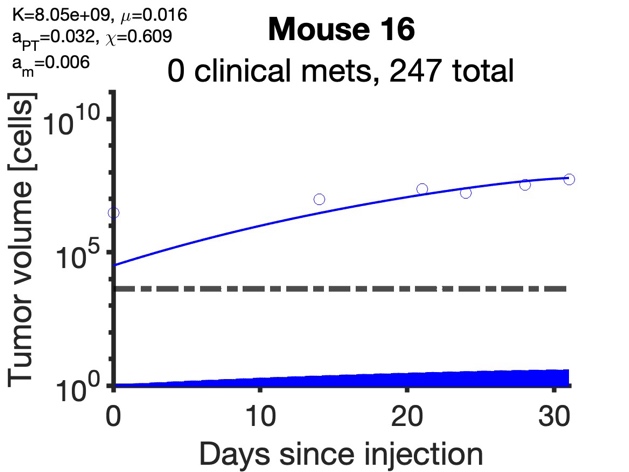


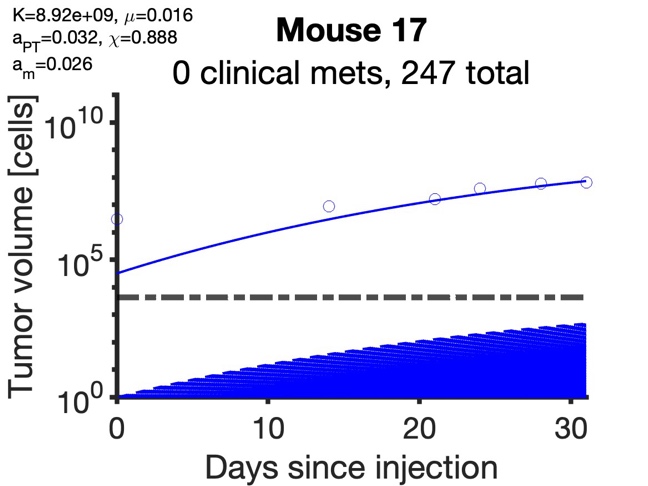

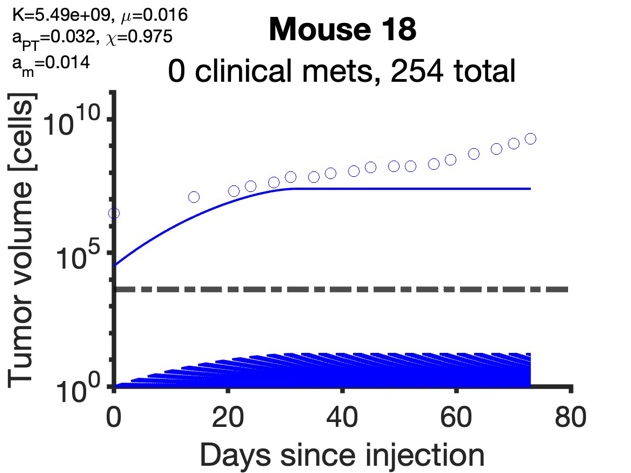


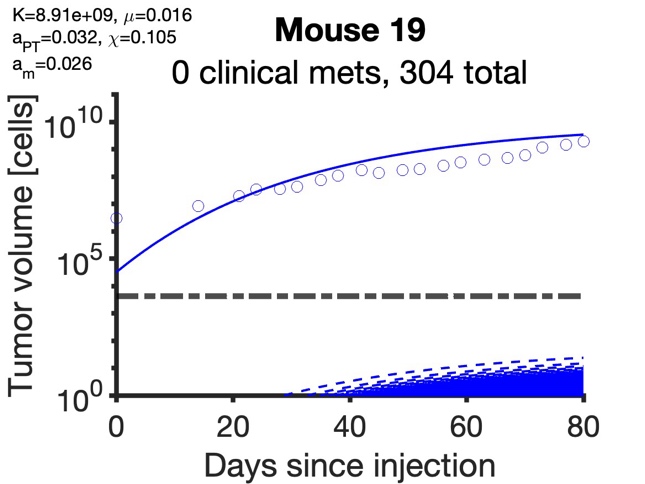


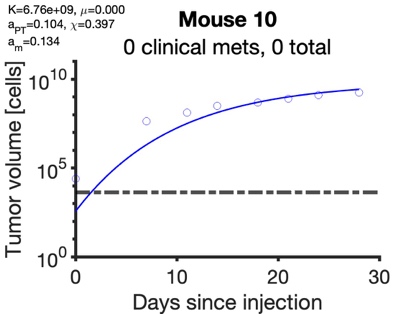

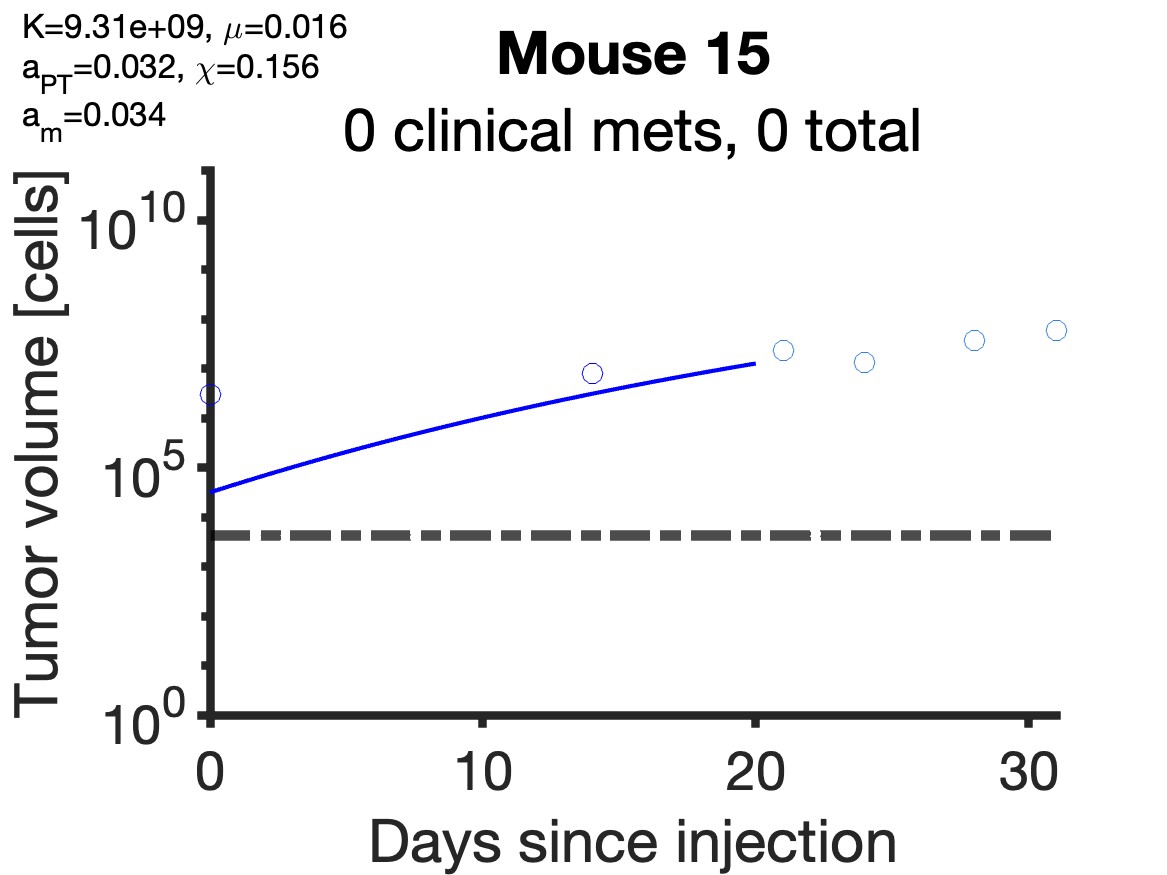

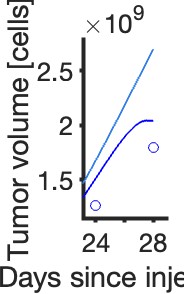


**Figure S2. *In silico* experiments.** No metastatic seeding for the mouse presented in panel 5A, i.e. $\mu=0$ **(A)**. We identified a larger volume of the primary tumor without seeding (upper curve) compared to the setting with seeding (lower curve) at the final day of simulated time. The primary tumor size simulation is shown on a linear scale in the callout window in orange boxes. This indicates a bi-directional concomitant systemic interconnectivity, as the presence of metastases inhibits the primary tumor growth as well. **(B)** We simulated a full surgical removal of the primary tumor at day 20 for another mouse of the Py230-C57BL/6 group and observe no effect on the metastatic disease manifestation. This indicates that the metastatic release after surgery may be a parameter-dependent effect.

**A**

**B**

**Two-tumor model with logistic growth**

The equations for the two-tumor model can easily be adjusted for logistic growth as

$\frac{dT_{1}}{dt}=r_{1}T_{1}\left( 1-\frac{T_{1}+T_{2}}{K} \right)$, $\frac{dT_{2}}{dt}=r_{2}T_{2}\left( 1-\frac{T_{1}+T_{2}}{K} \right)$

with the initial conditions $T_{1}\left( 0 \right)=T_{1,0}$ and $T_{2}\left( 0 \right)=T_{2,0}$. The derived properties when equal growth rates are assumed (i.e., $r_{1}=r_{2}$) still hold. Below are the fits towards respective data from the ST and DT groups, and their normalized root mean square error. The performance is worse than for Gompertzian growth, as presented in the manuscript.

**Figure S3. Model training to the mice in the single and double tumor experiment for logistic growth.** The individual fits and residuals towards the data of the first experiment of **(A)** the ST group (blue) and **(B)** the DT group (larger tumor: blue; smaller tumor: orange) for the parsimonious model formulation identified by the minimum BIC, but with logistic growth featuring shared carrying capacity and equal intrinsic growth rate for both tumors in the DT group. The residual plots show the normalized root mean square error (NRMSE) for the fits.


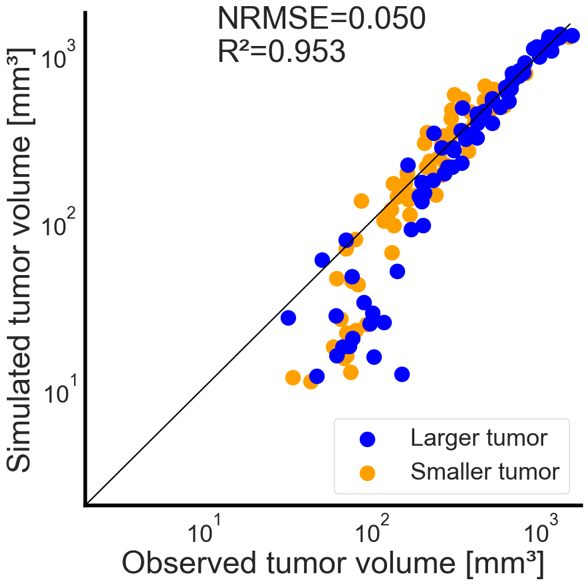

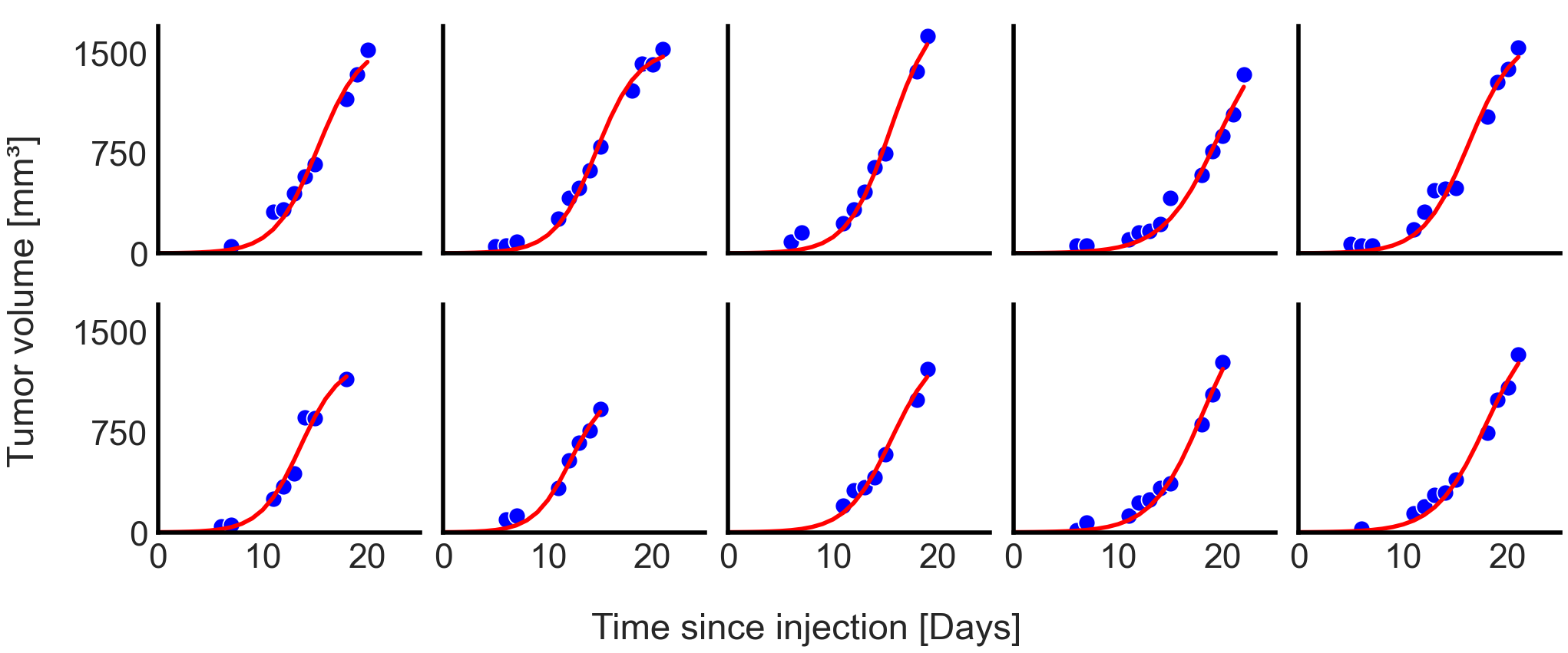

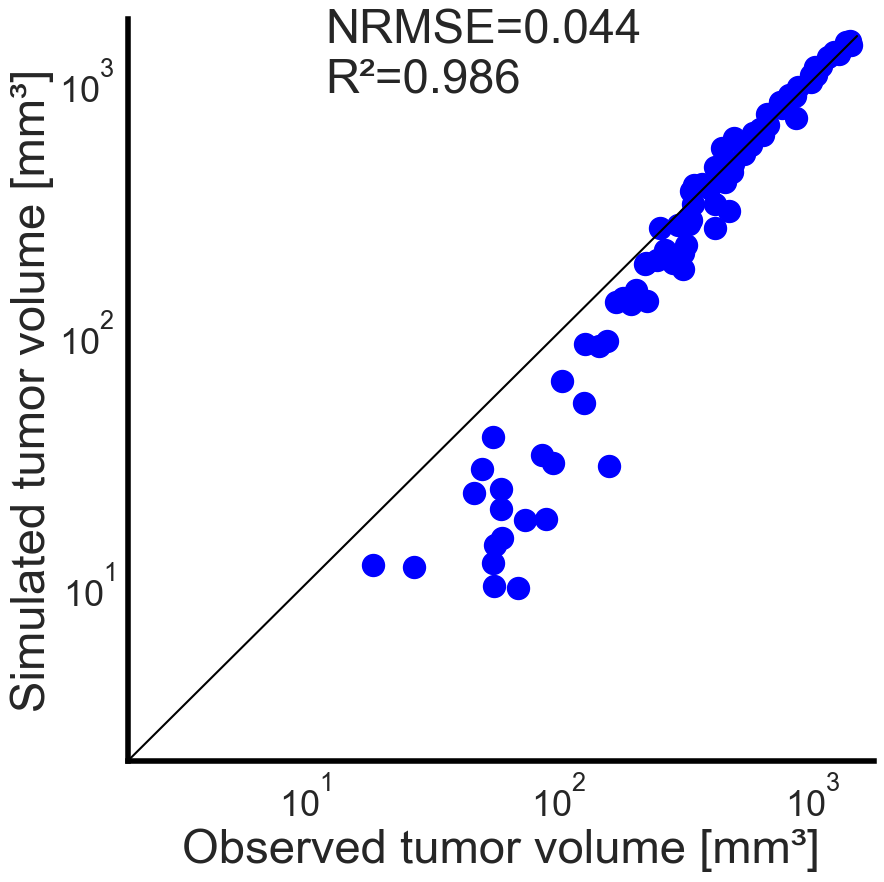

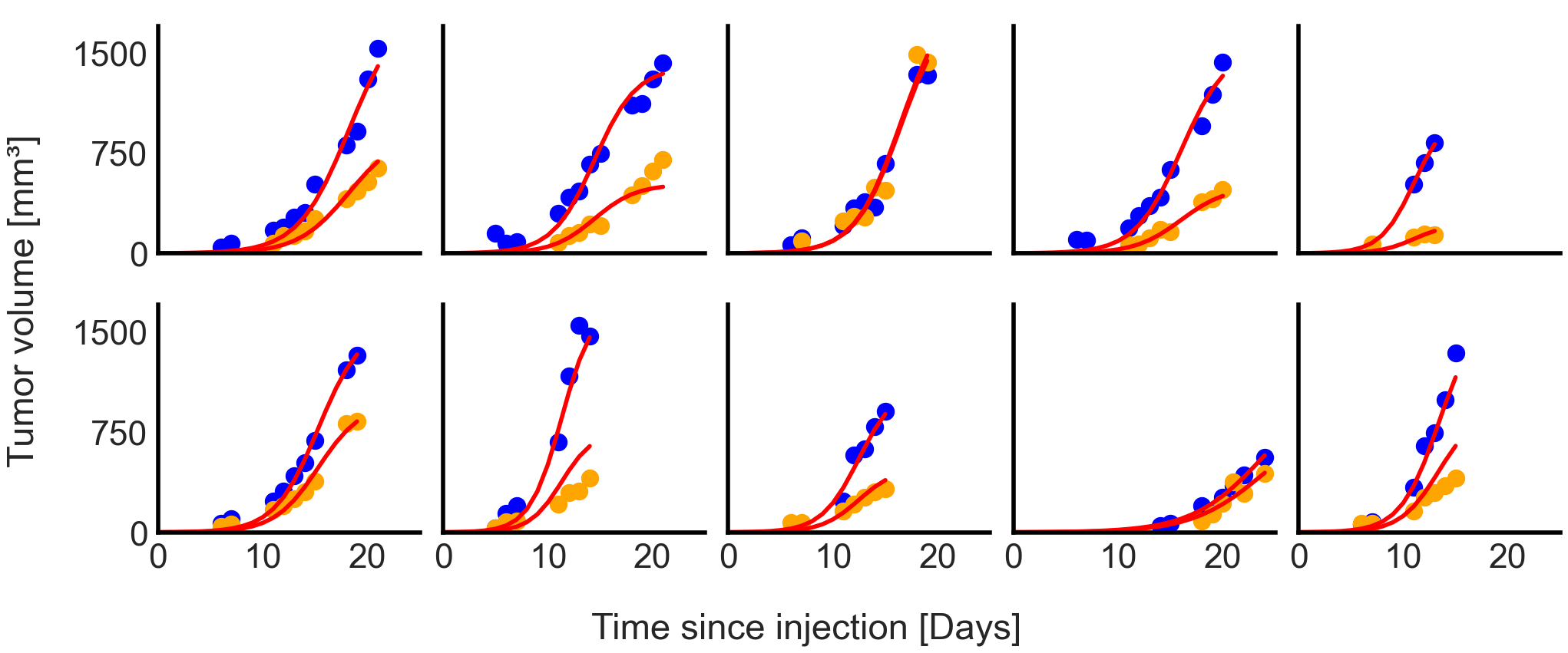


**A**

**B**

**Exponential growth models for comparative purpose**

To highlight the model advantages of and preference towards a shared carrying capacity, we fitted the data for the ST and DT groups to exponential growth models. In case of the ST group, we used the growth equation $\frac{dT_{1}}{dt}=r_{1}T_{1}$, for the DT group we used the system of ordinary differential equations $\frac{dT_{1}}{dt}=r_{1}T_{1}, \frac{dT_{2}}{dt}=r_{2}T_{2}$. The initial conditions are $T_{1}\left( 0 \right)=T_{1,0}$ and $T_{2}\left( 0 \right)=T_{2,0}$ (the latter only for the DT group). The NRMSE values are worse than for logistic or Gompertz growth, supporting the concept of a shared carrying capacity.

**Figure S4. Model training to the mice in the single and double tumor experiment for exponential growth.** The individual fits and residuals towards the data of the first experiment of **(A)** the ST group (blue) and **(B)** the DT group (larger tumor: blue; smaller tumor: orange) for a model formulation using one (ST) or two (DT) exponential growth curves $\frac{dT(t)}{dt}=rT(t)$. The residual plots show the normalized root mean square error (NRMSE) for the fits.


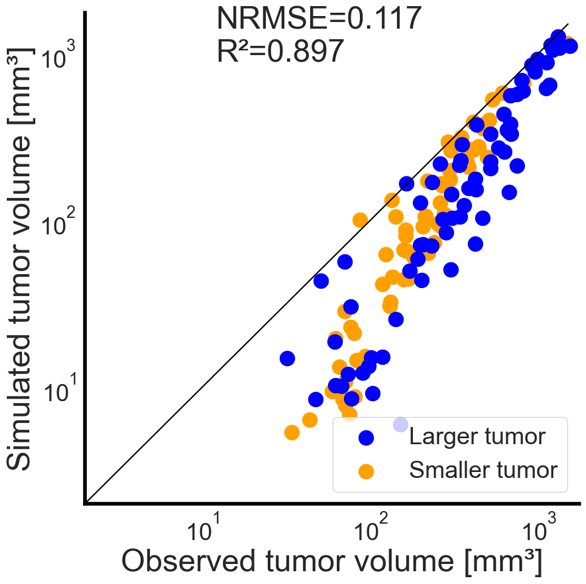

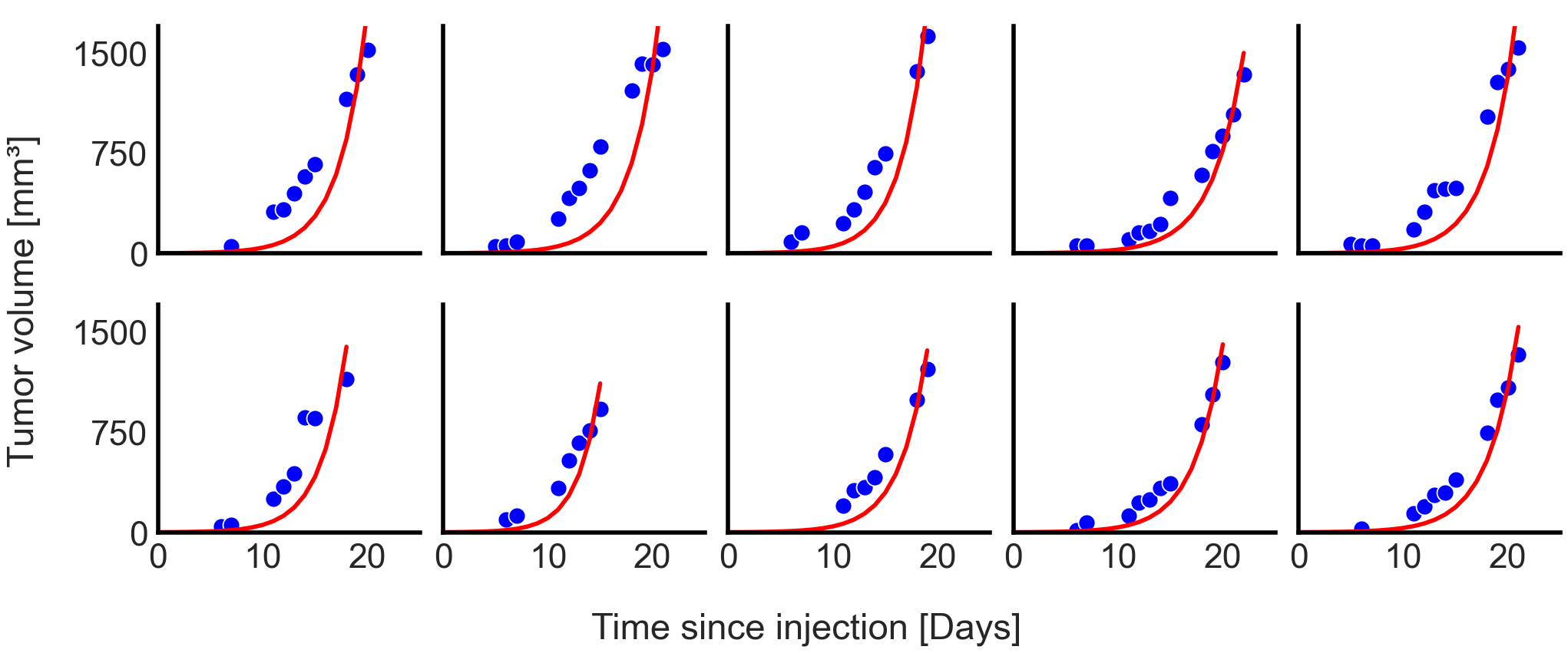

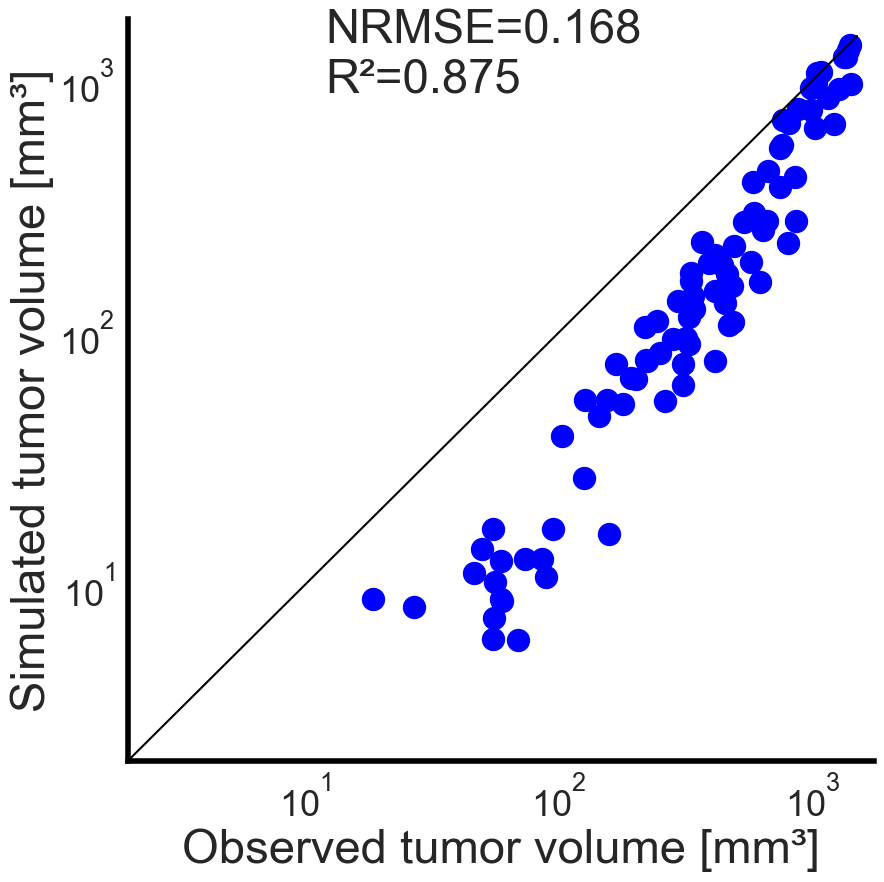

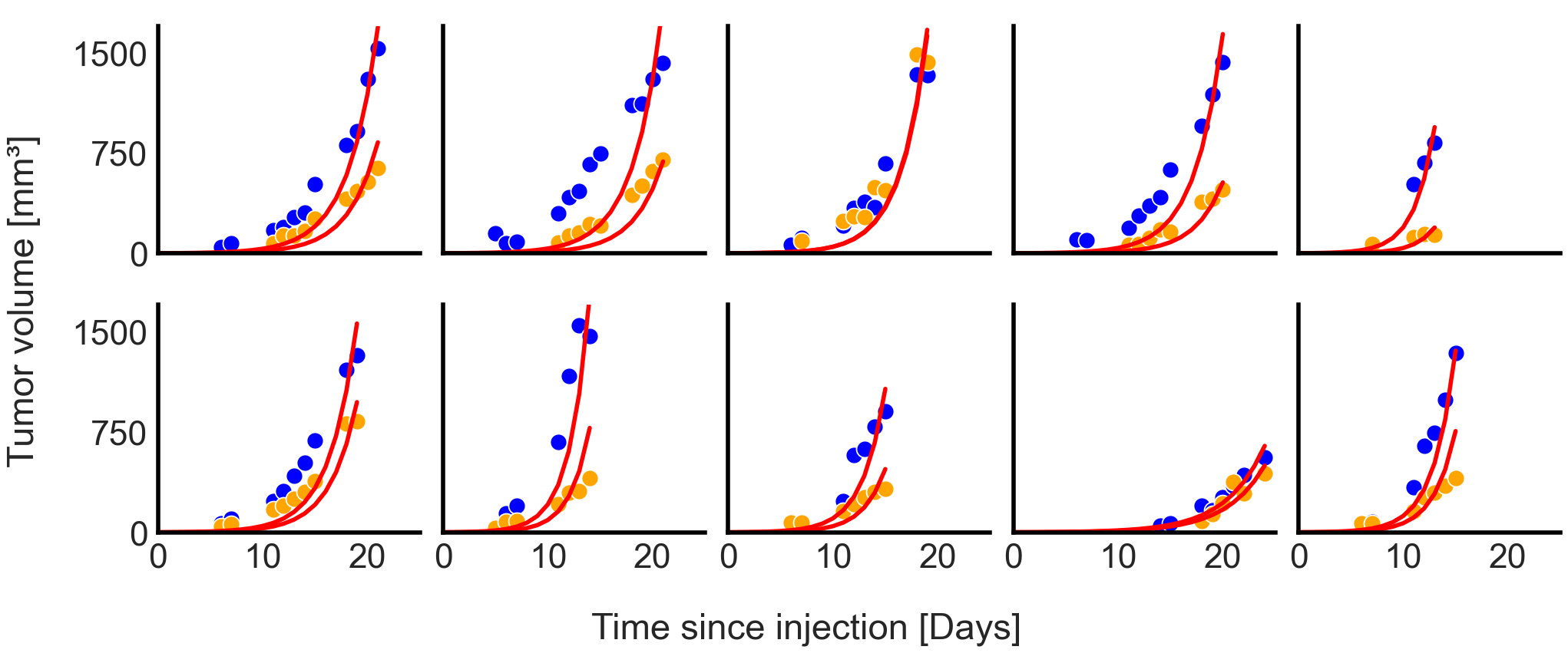


**A**

**B**


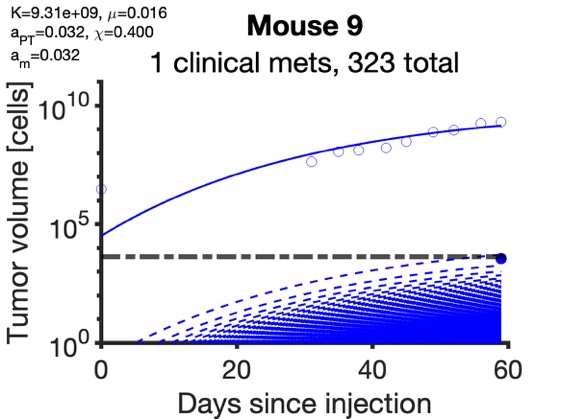

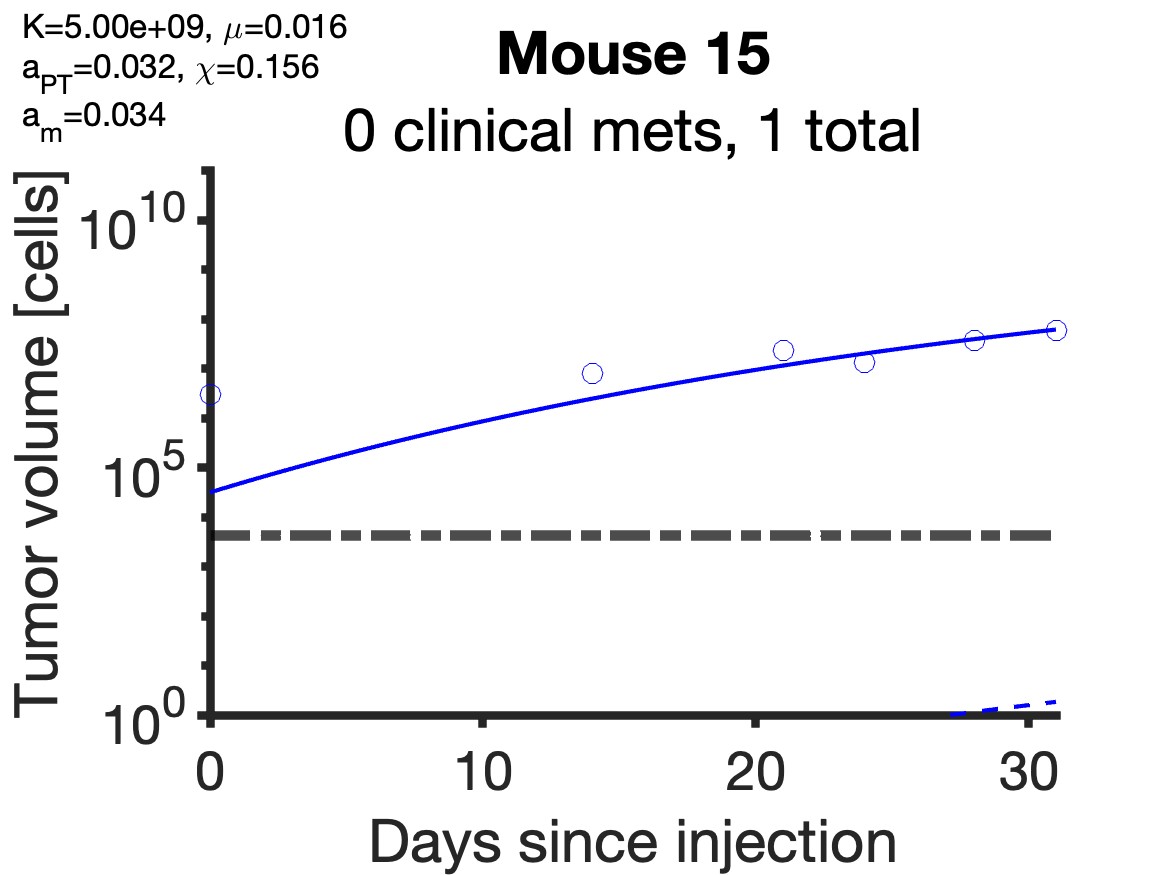


**Figure S5. *In silico* experiments.** Surgery-free simulations of mouse 9 and 15, but with swapped carrying capacity parameter $K$. The simulations do not differ greatly from the original simulation. This suggests that the qualitative manifestation of metastatic disease is regulated by the parameters $a_{m}$ and $\chi$.

**A**

**B**


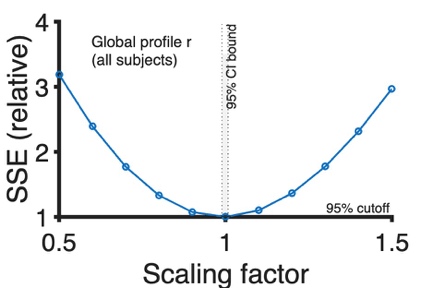

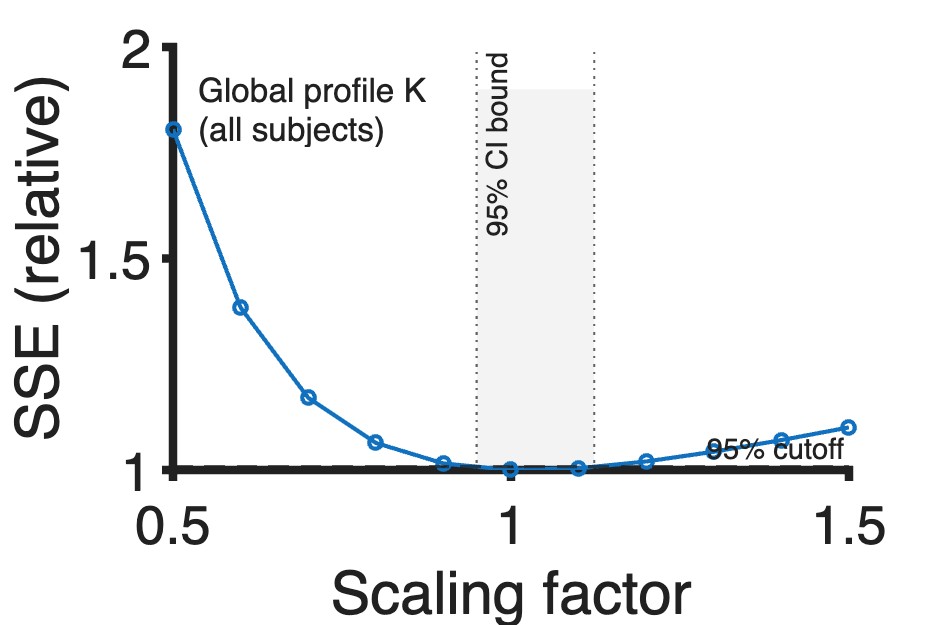


**Figure S6. *Parameter identifiability*.** These plots show the profile loglikelihoods estimated through the profile SSE for the two-tumor model **(A)** and the tumor-metastases model for group 4T1-BALB/c **(B)** and Py230-C57BL/6 **(C)**. For the latter one, we neglected mice that did not show metastases at time point of sacrifice. Horizontal dashed lines indicate the 95% likelihood-ratio cutoff on relative SSE, and shaded bands mark the respective 95% confidence region on the scaling factor. The shared carrying capacity relatively exhibits one-sided 95% bounds, but all other parameters show strong curvatures and good identifiability.

**A**


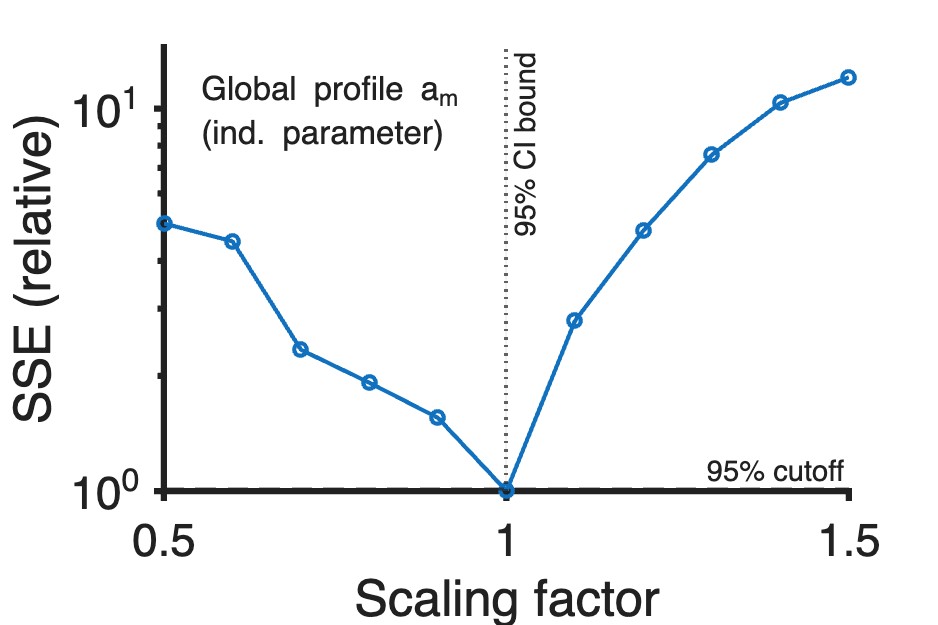

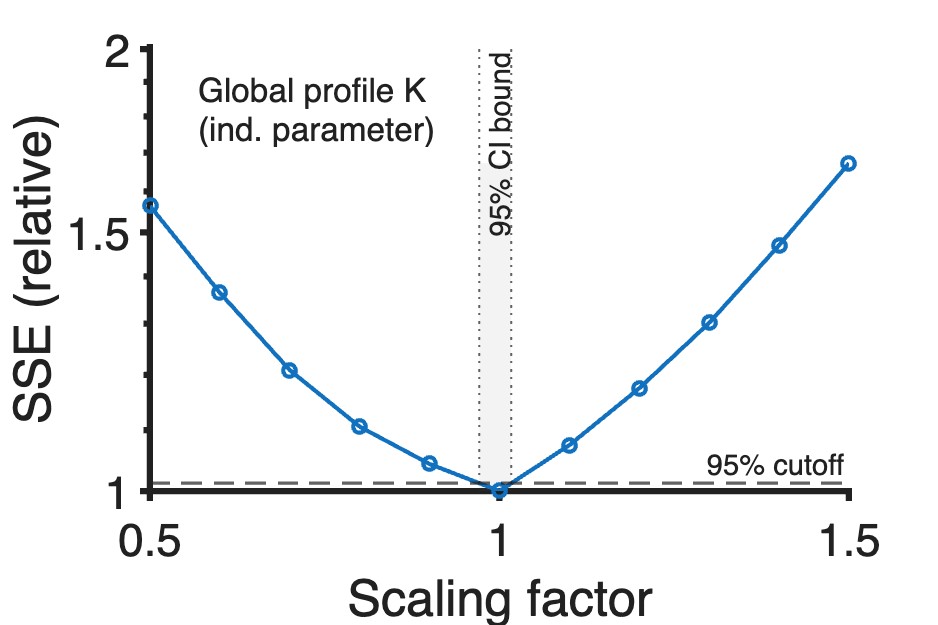


**B**


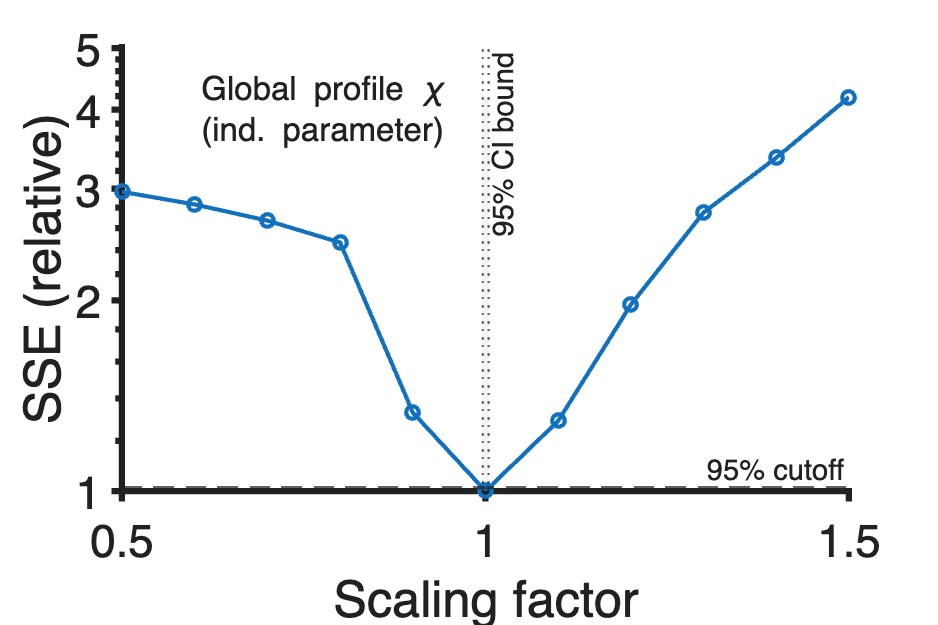

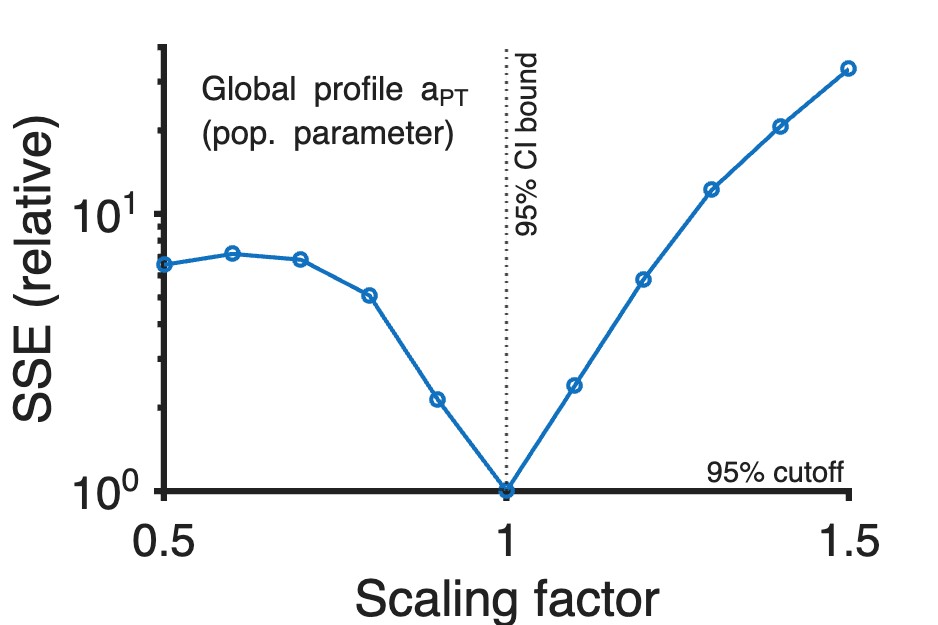

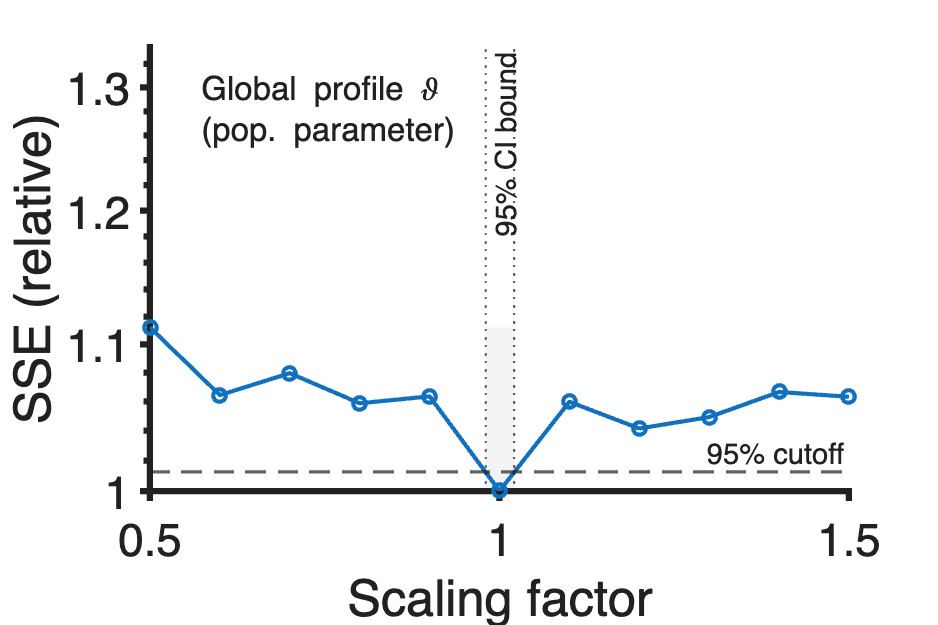

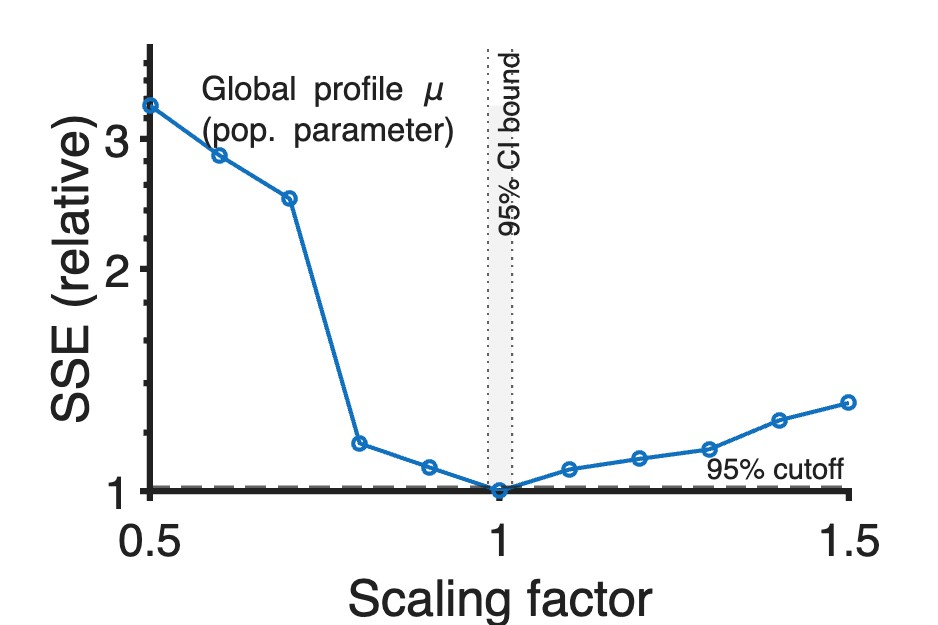

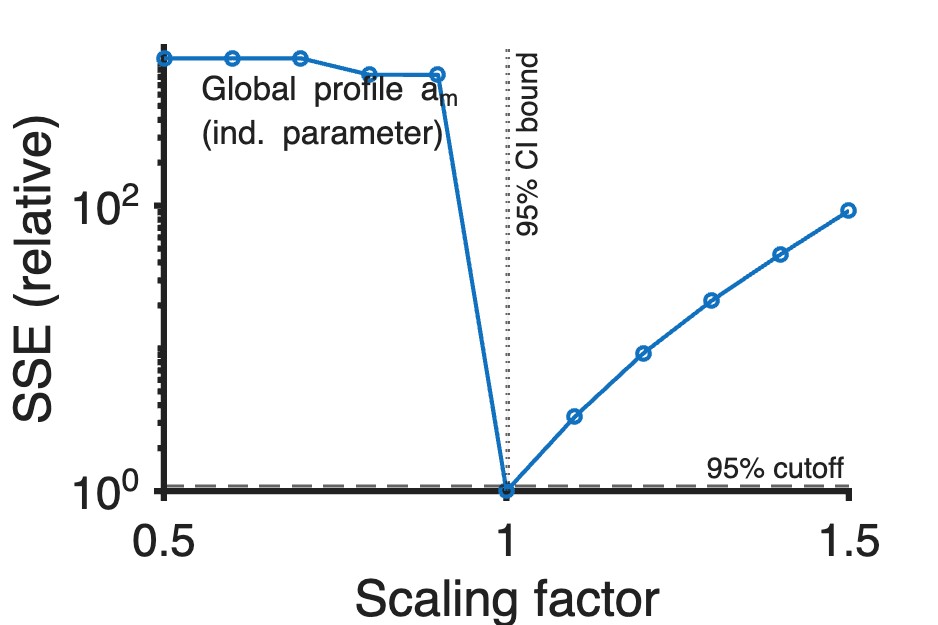

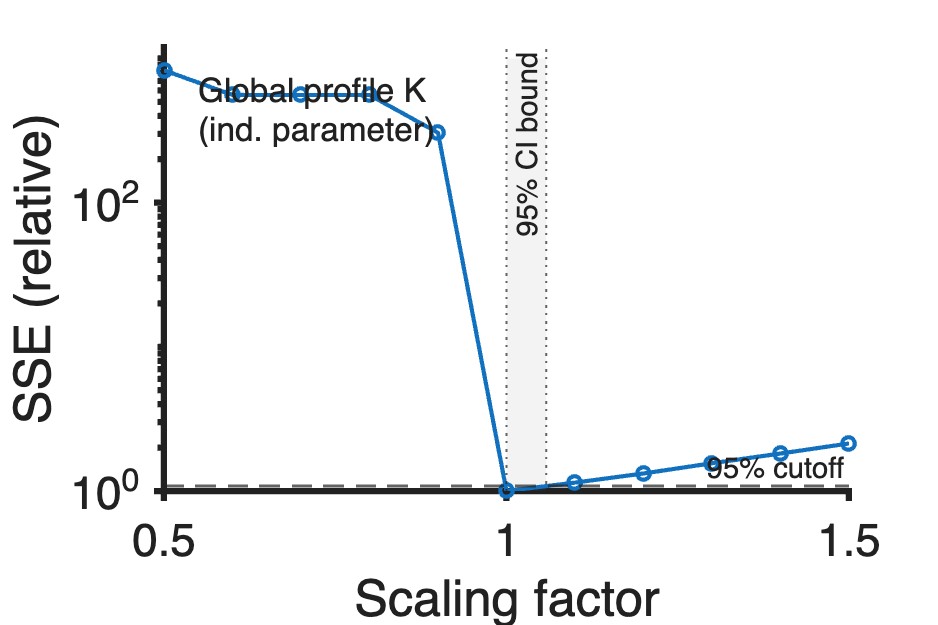


**C**


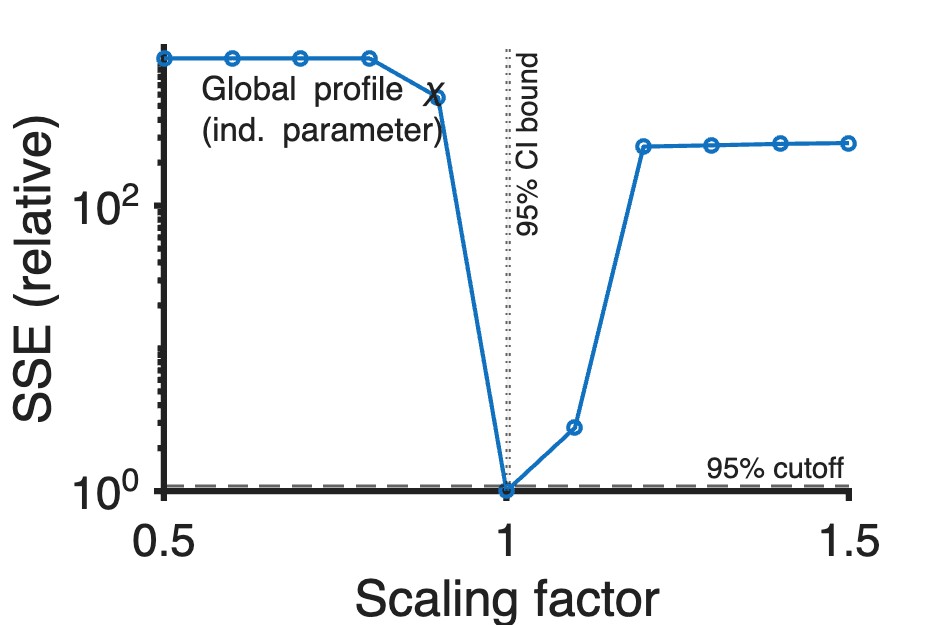

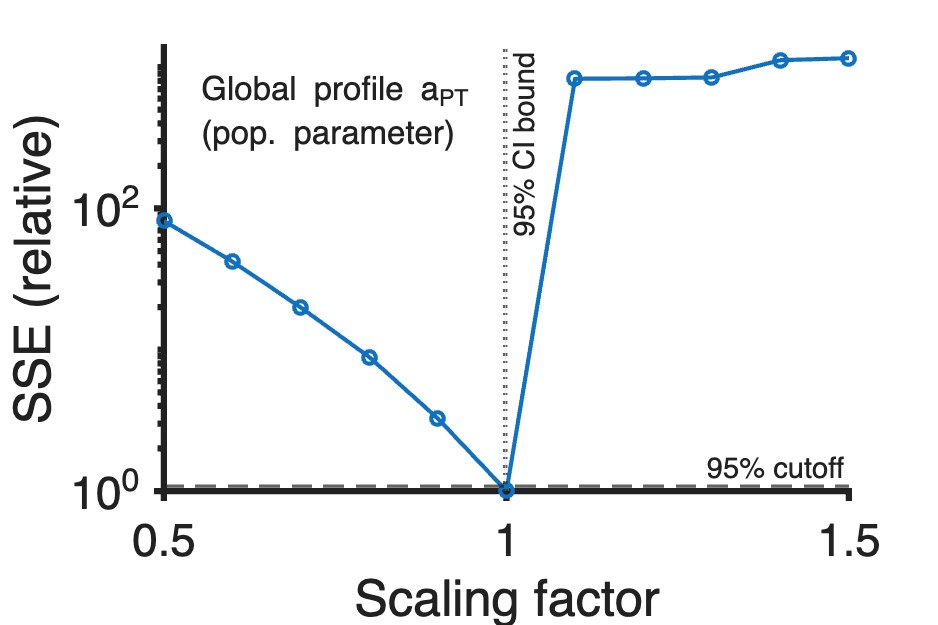

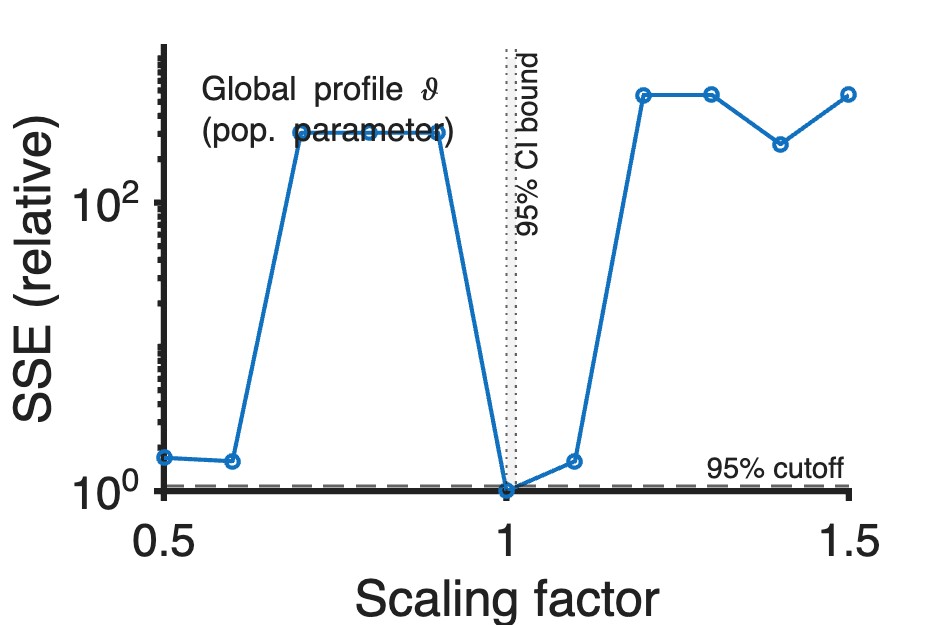

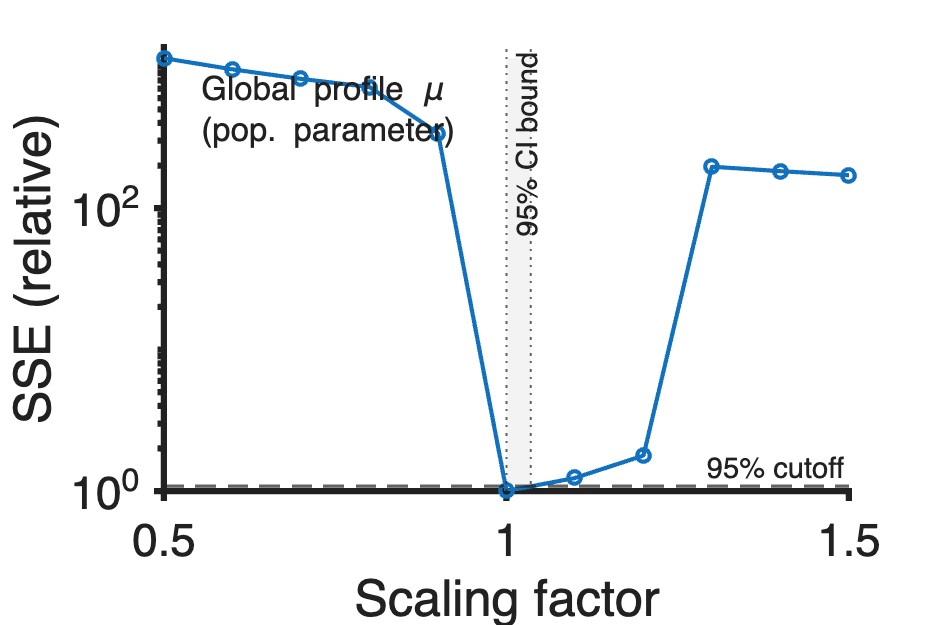


**Figure S7. *Parameter perturbation effects on number of metastases for the* 4T1-BALB/c group.** These plots show the relative effects of parameter perturbations on the number of **(A)** total and the **(B)** clinically relevant metastases. Perturbations within the 95% CI bounds from Figure S6 preserve the qualitative change in number of metastases.


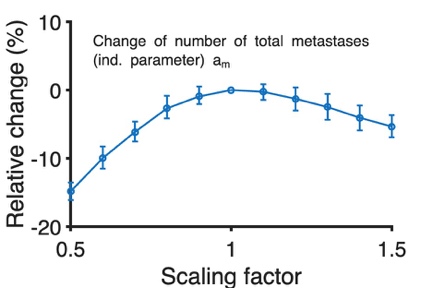

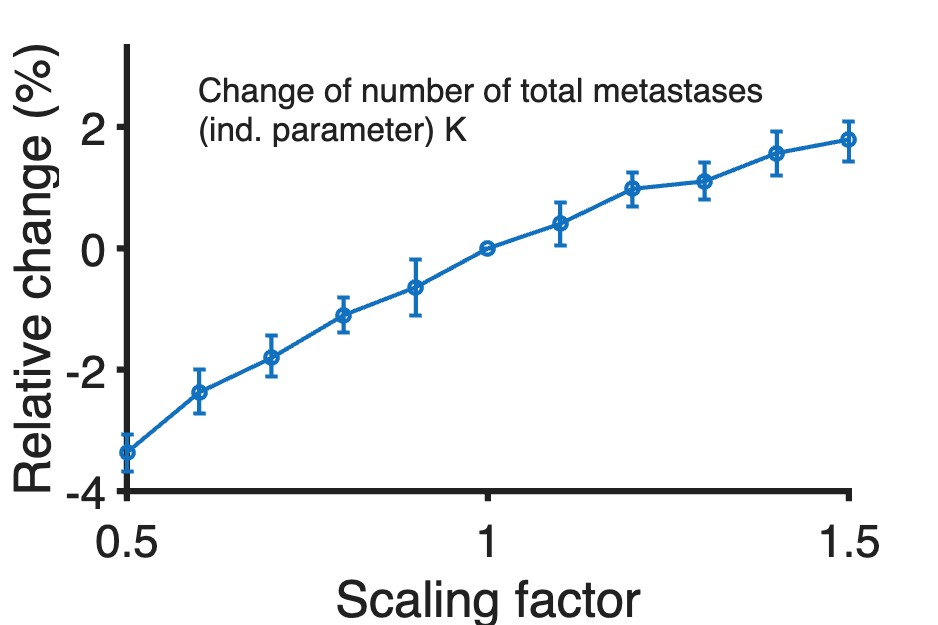


**A**


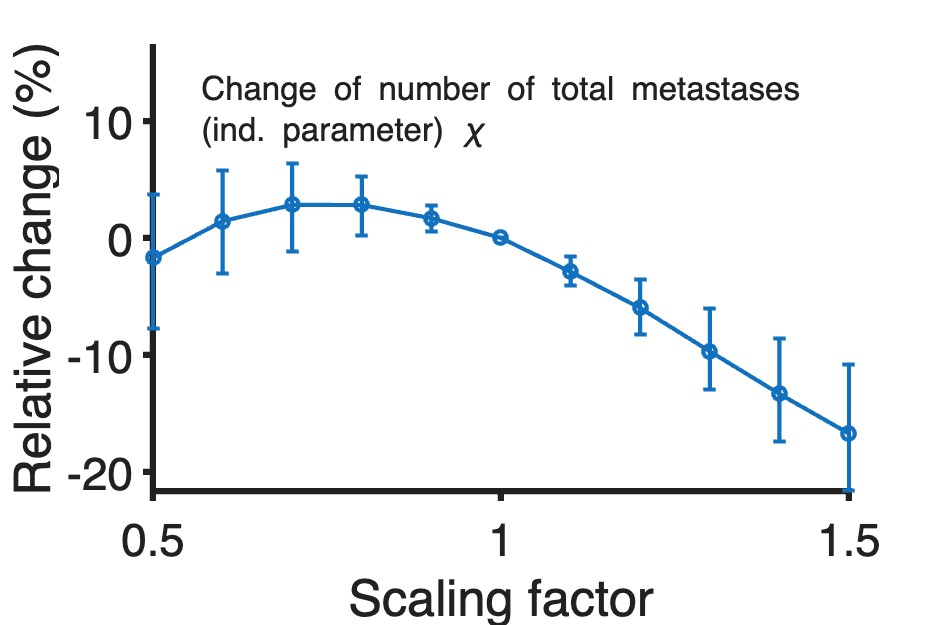

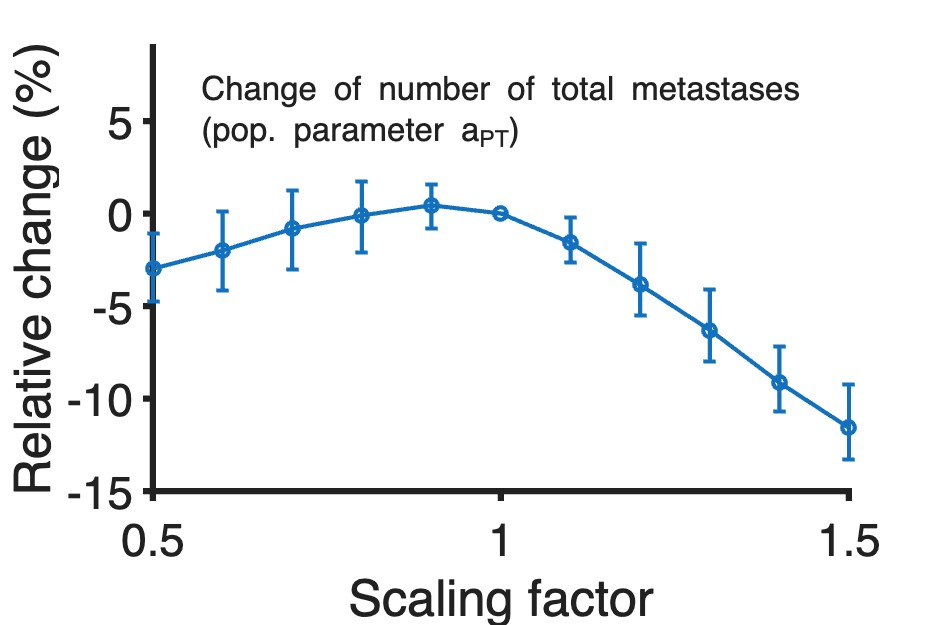

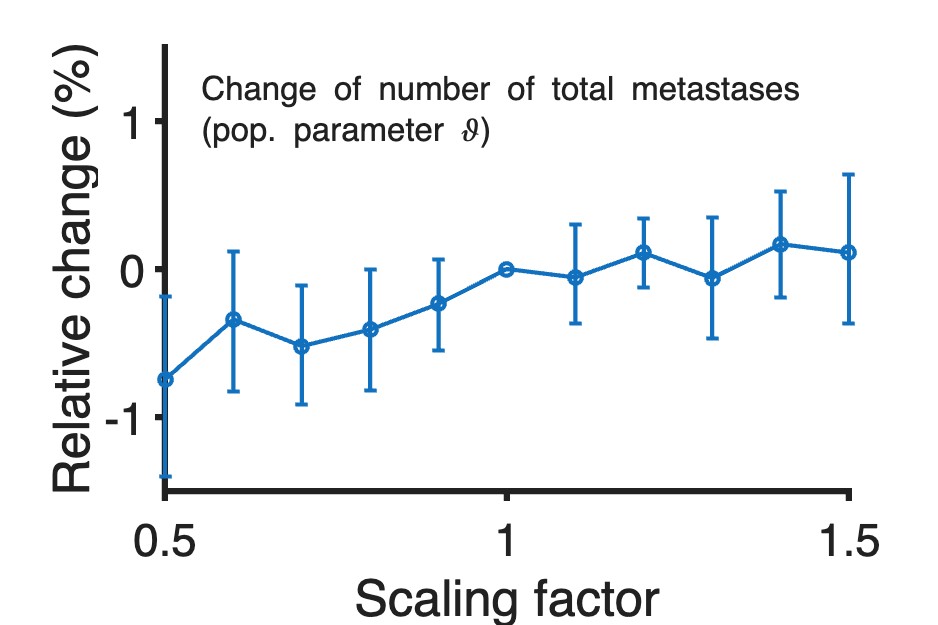

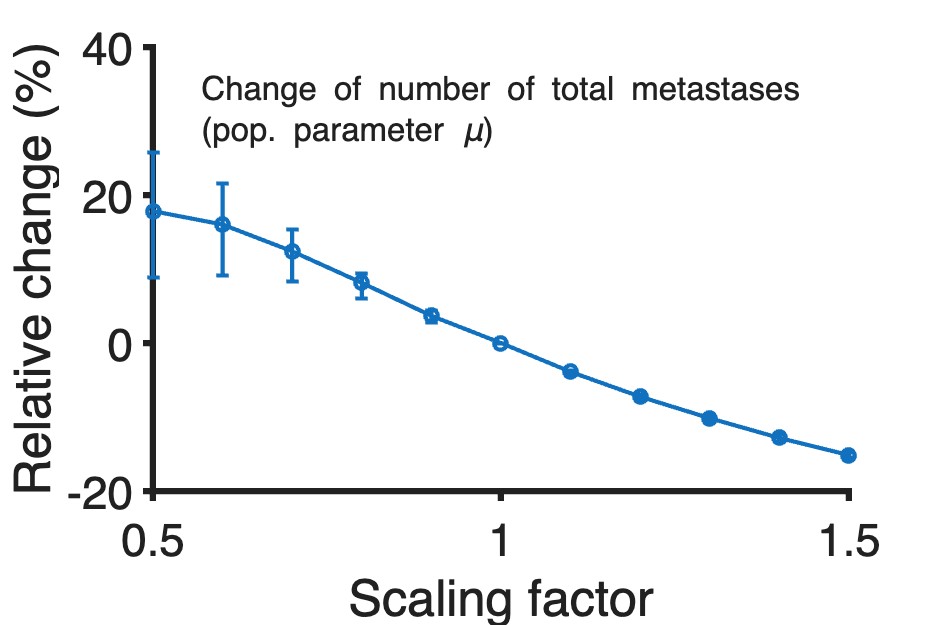

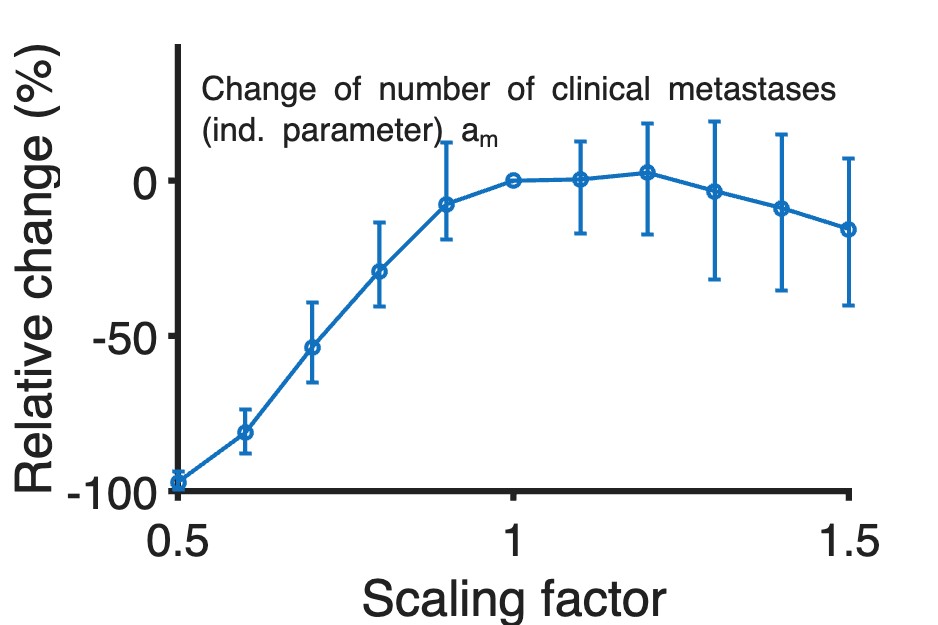

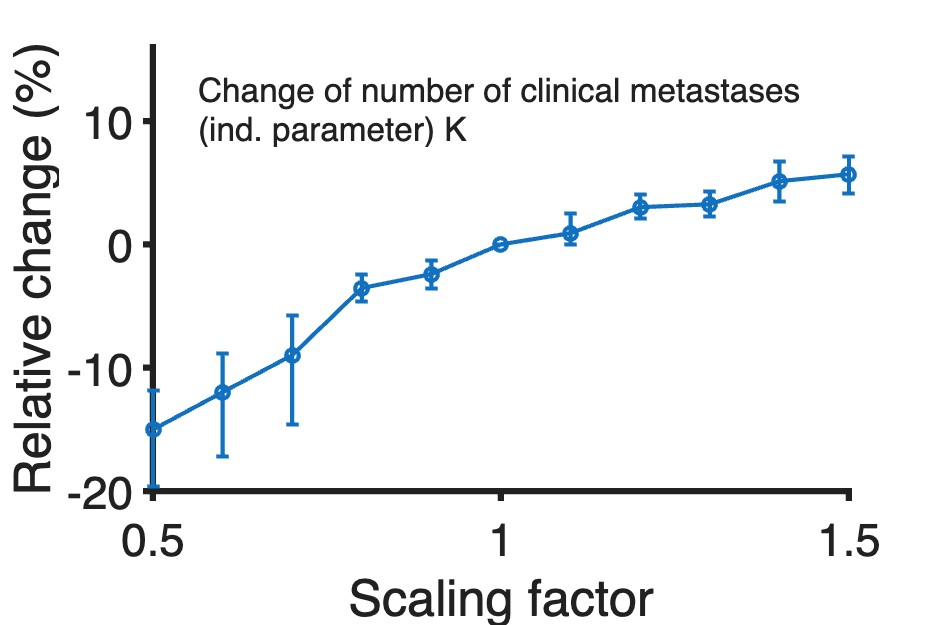


**B**


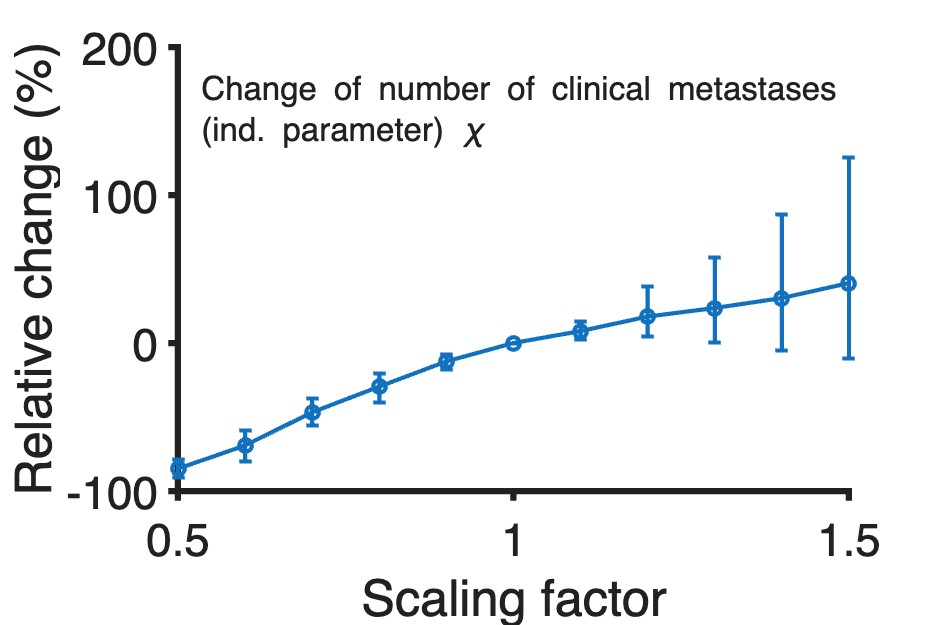

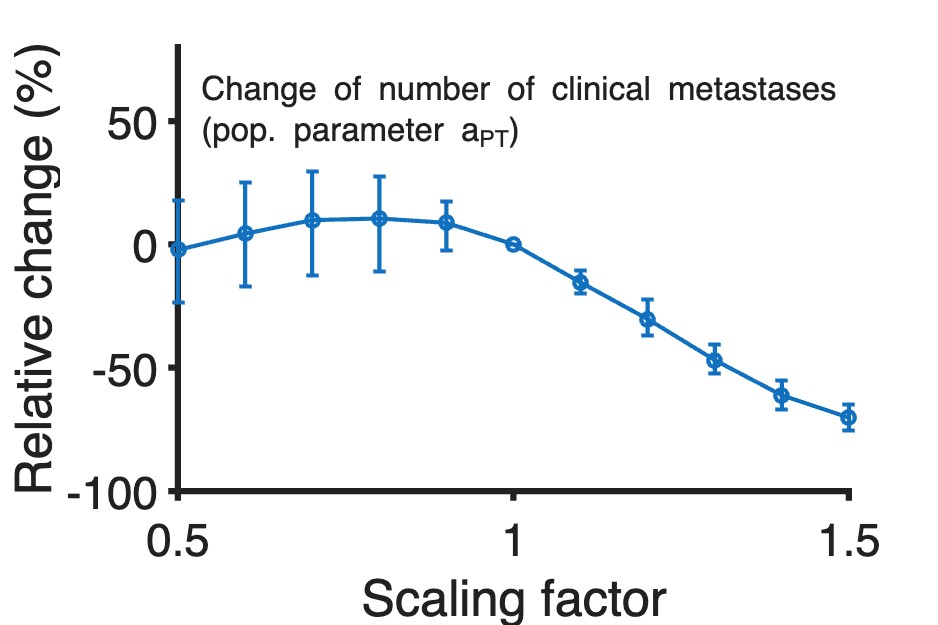

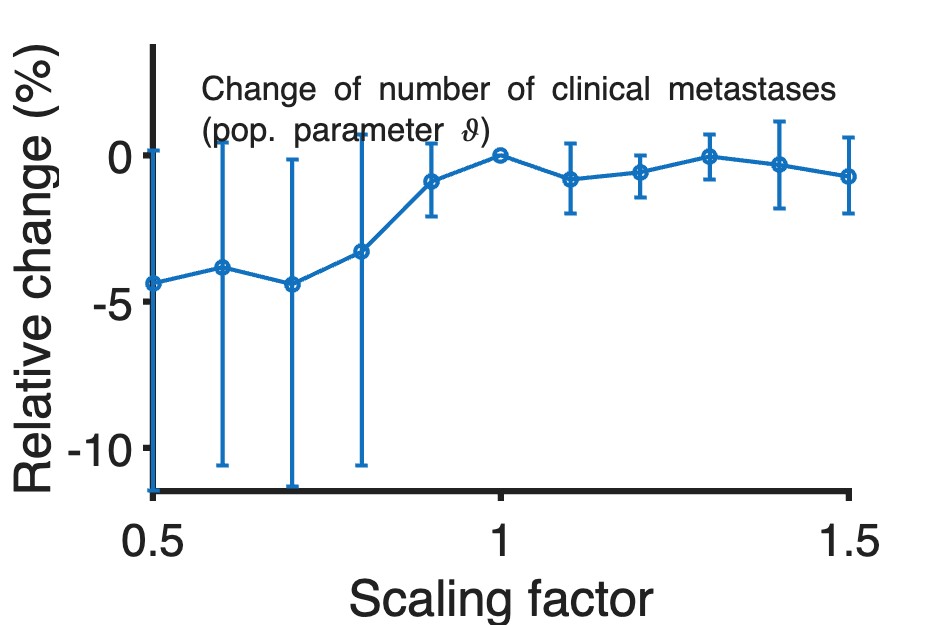

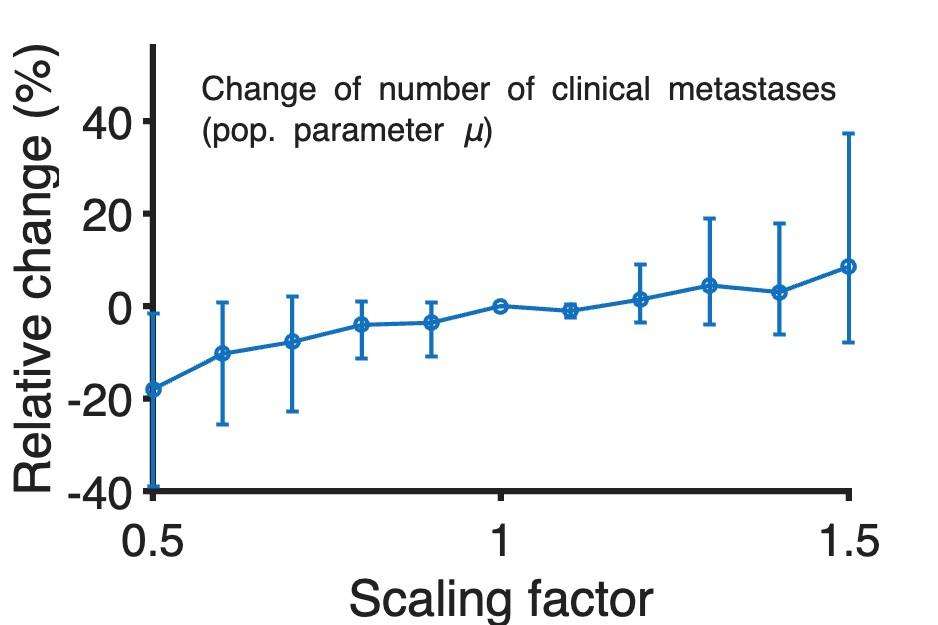


**Figure S8. *Parameter perturbation effects on number of metastases for the* Py230-C57BL/6 group.** These plots show the relative effects of parameter perturbations on the number of **(A)** total and the **(B)** clinically relevant metastases. For this analysis we neglected mice that did not show metastases at time point of sacrifice. Perturbations within the 95% CI bounds from Figure S6 preserve the qualitative change in number of metastases.


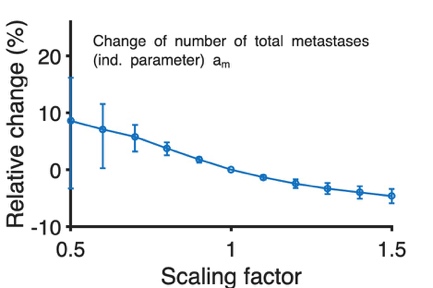

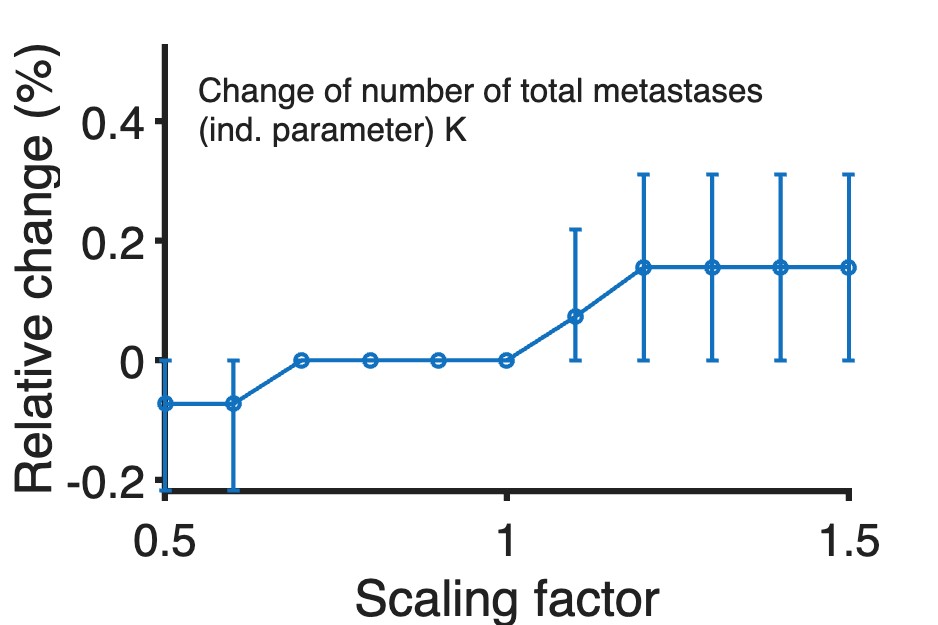


**A**


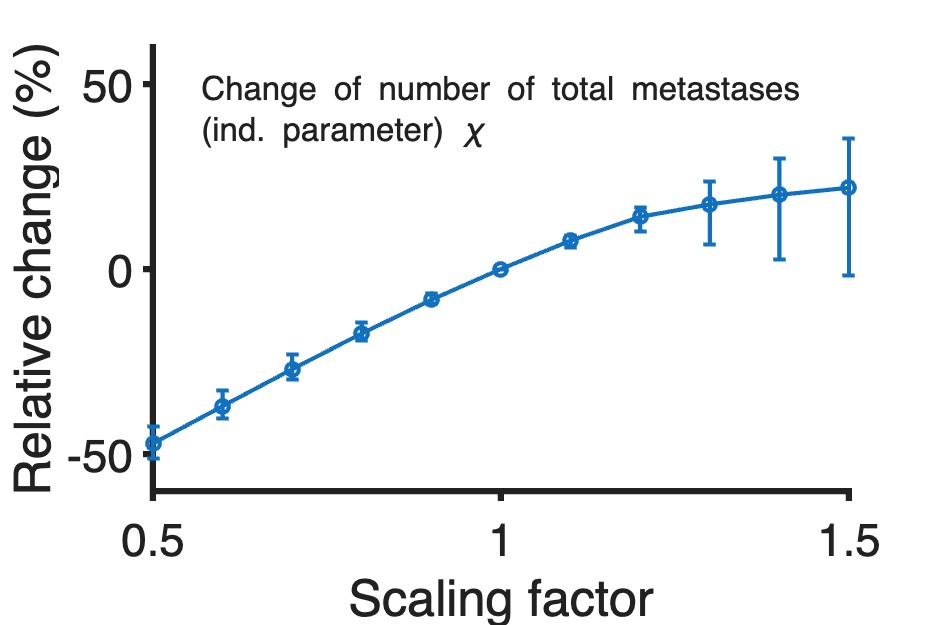

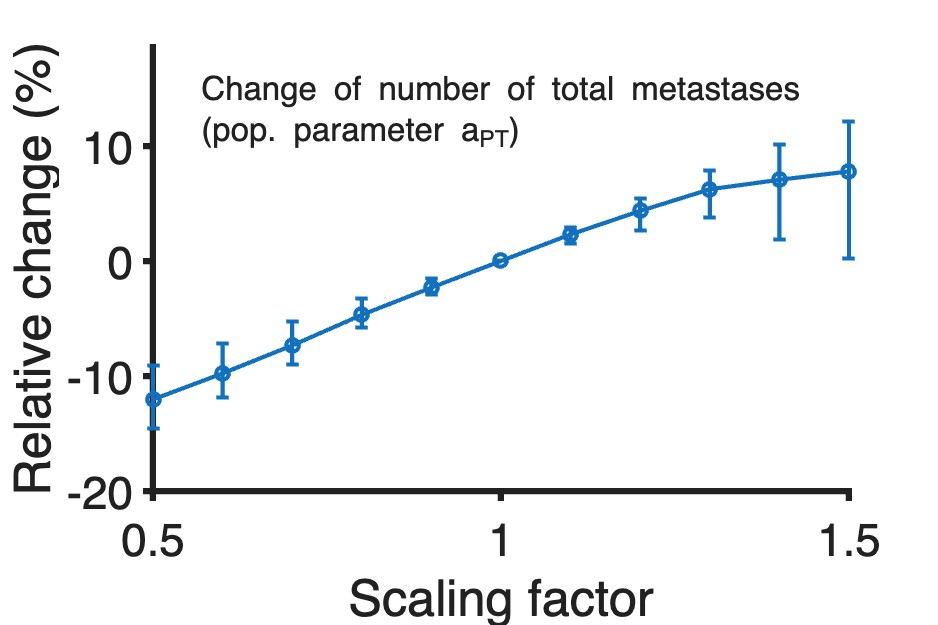

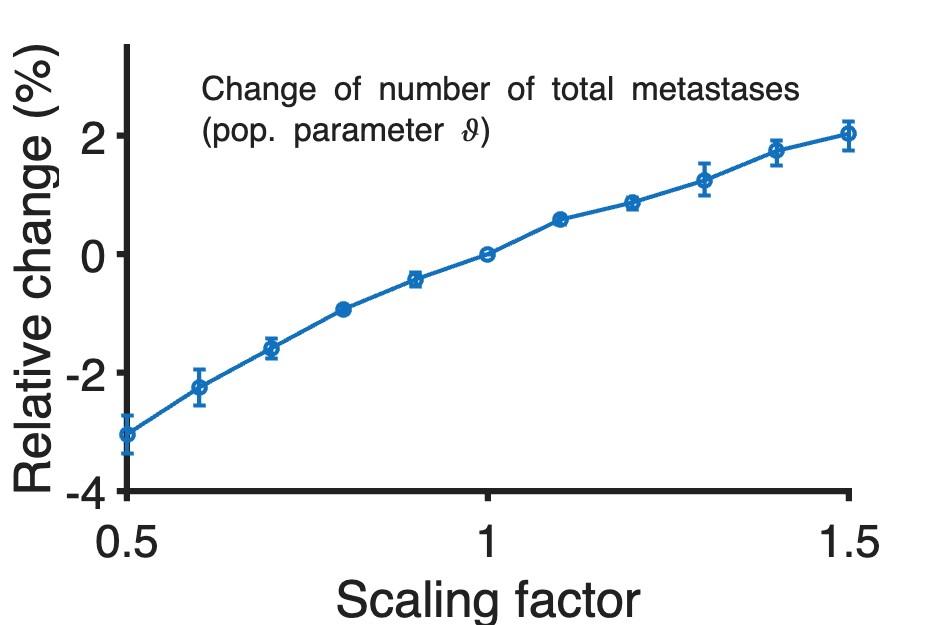

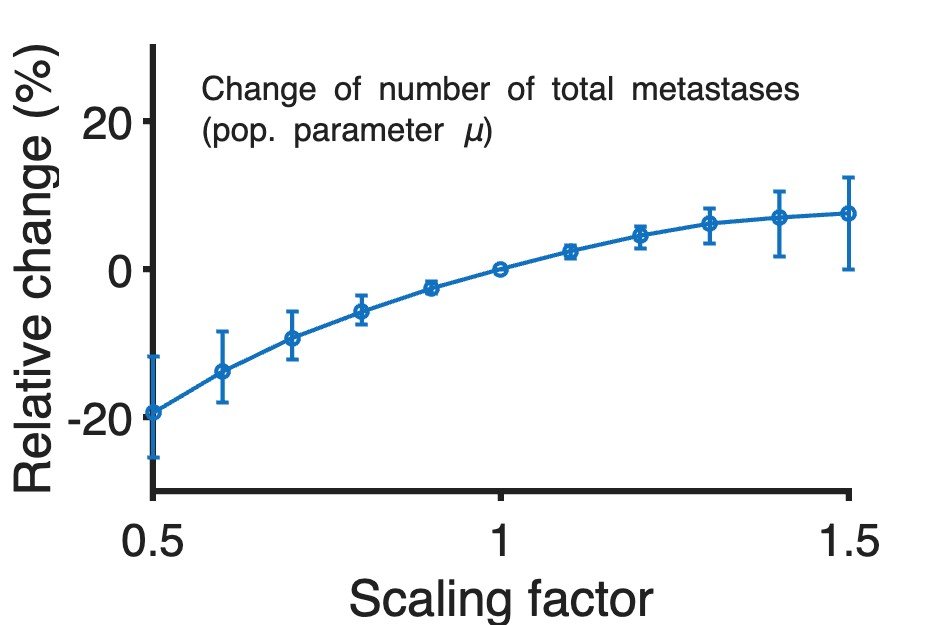

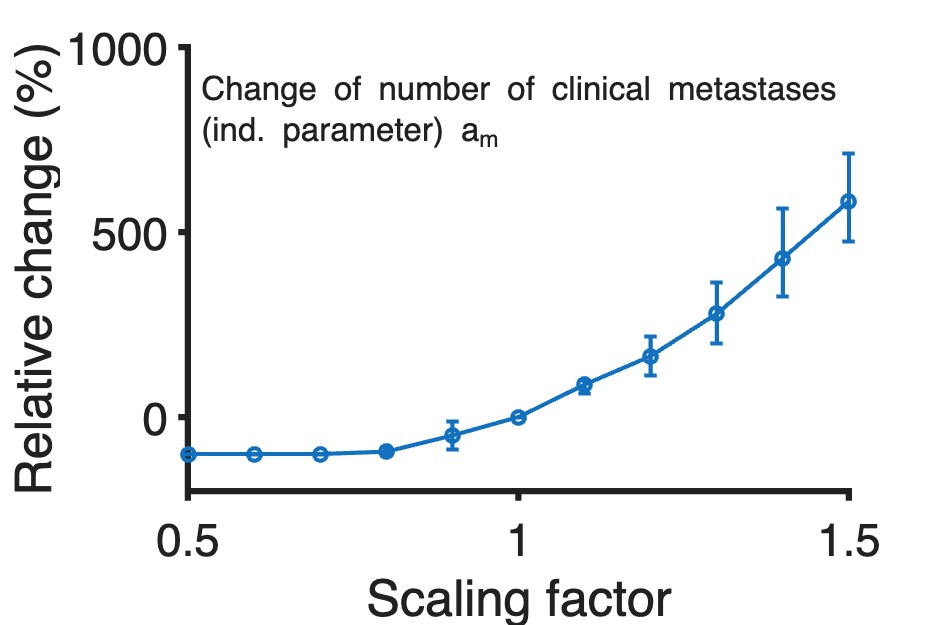

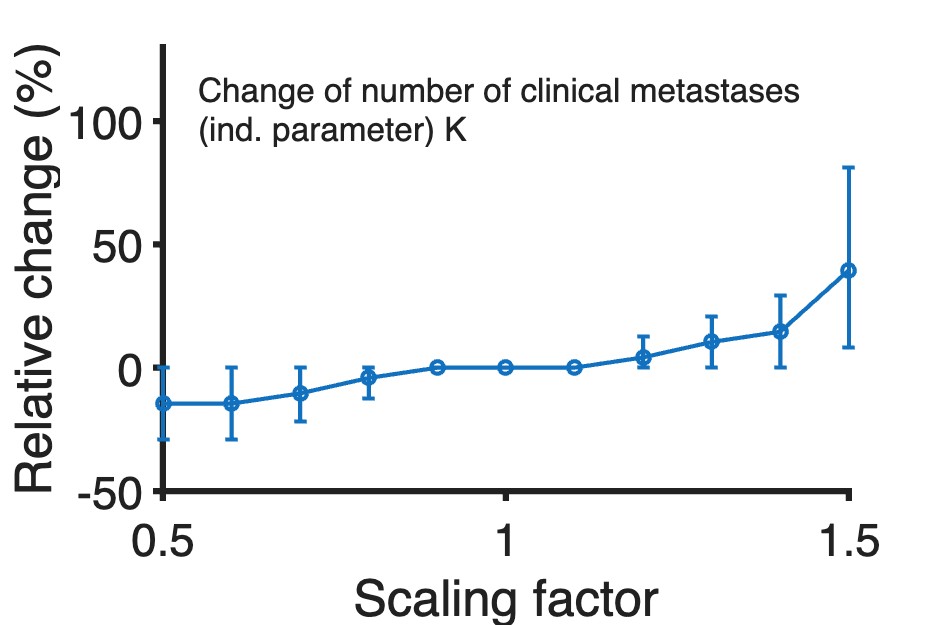


**B**


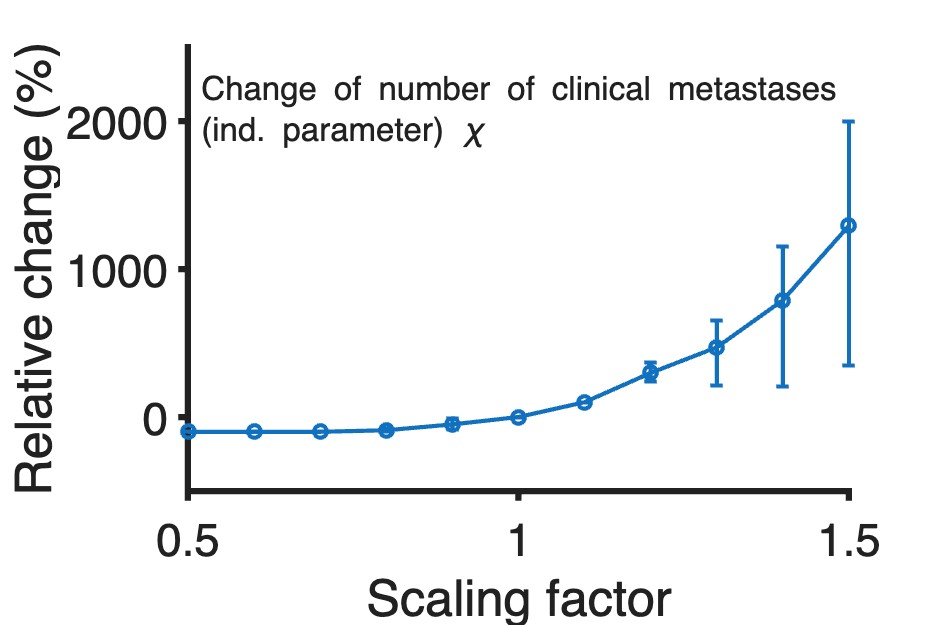

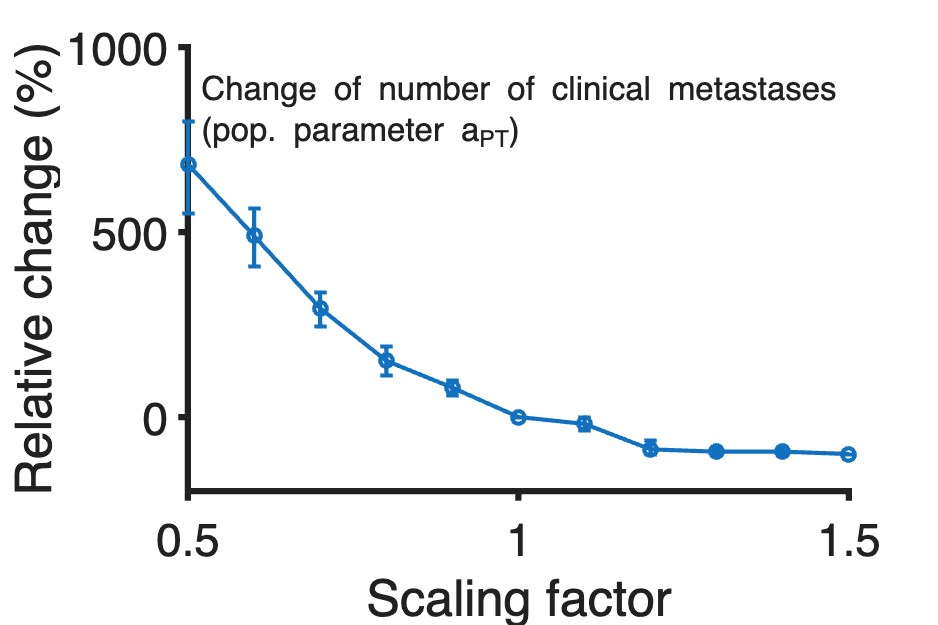

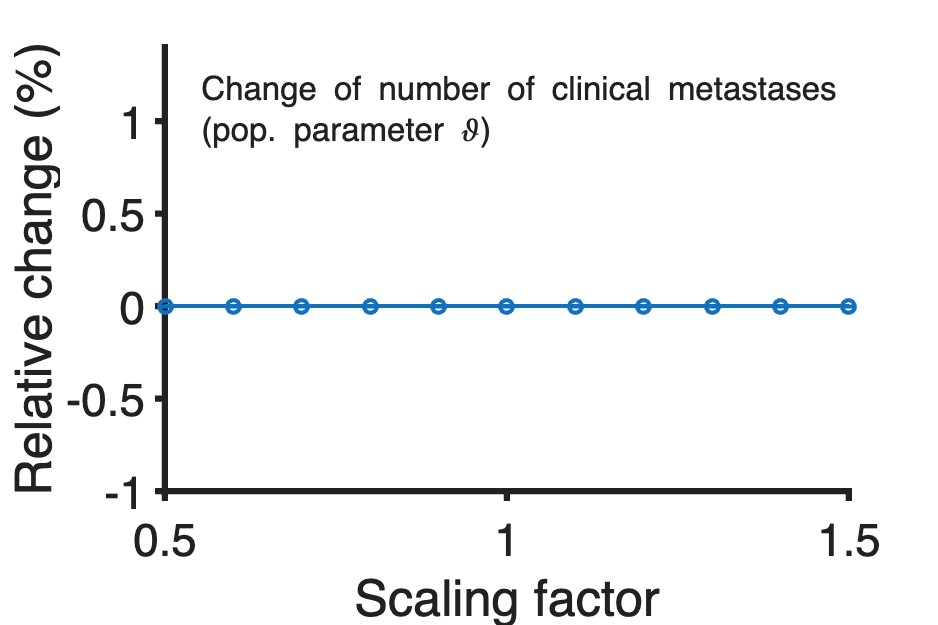

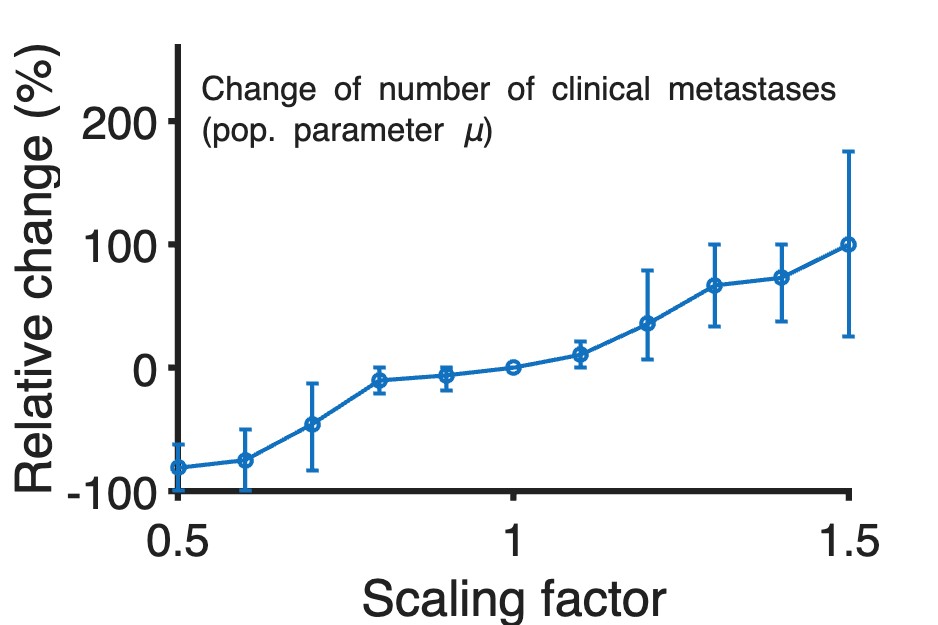


**Figure S9. *Parameter perturbation effects after hypothetical surgery on number of metastases for the* 4T1-BALB/c group.** These plots show the relative effects of parameter perturbations on the number of **(A)** total and the **(B)** clinically relevant metastases after hypothetical surgery at day 20. The metastatic growth rate $a_{m}$ and the cell propensity for metastasis $\chi$ are the two parameters mainly driving presence of metastases.


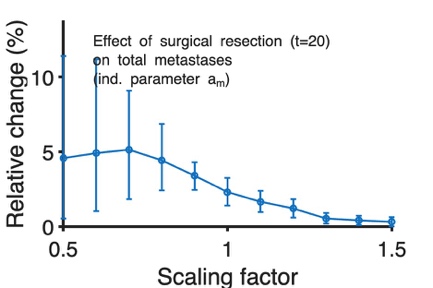

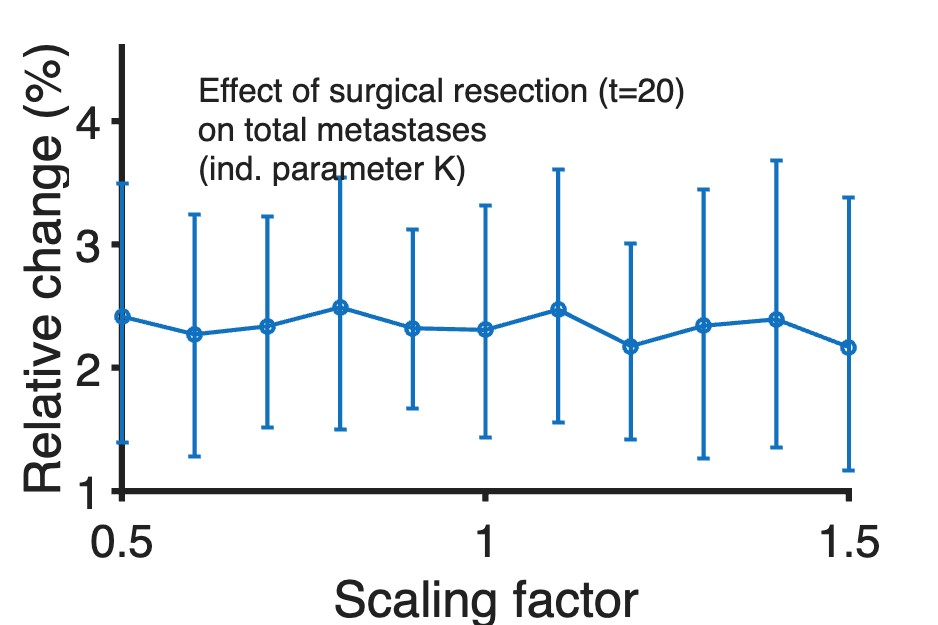


**A**


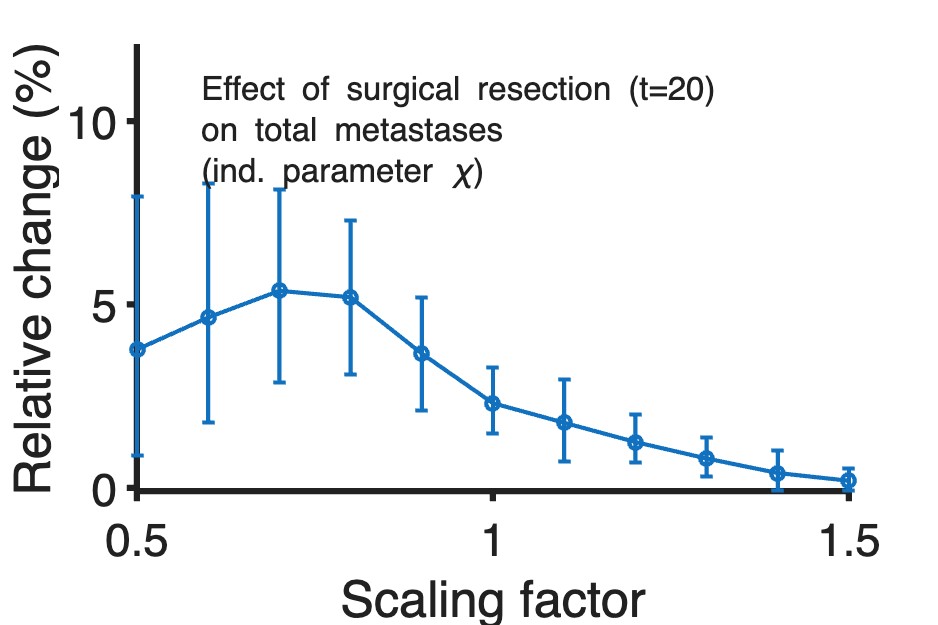

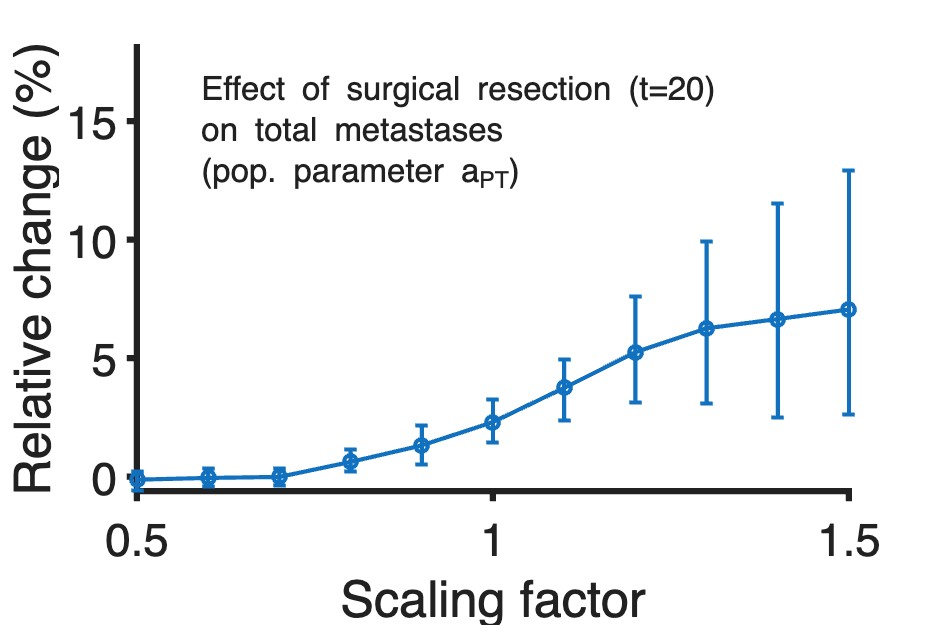

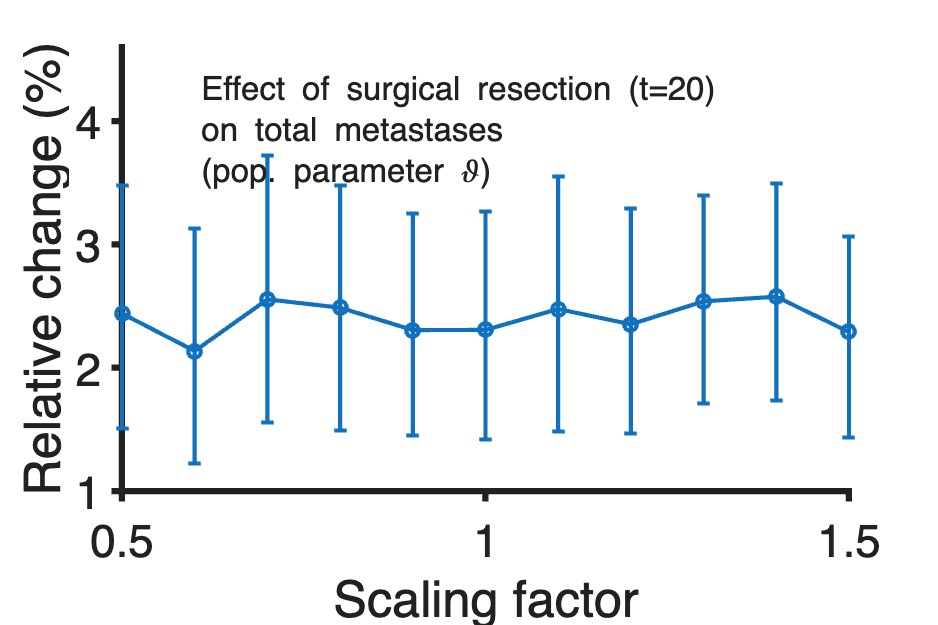

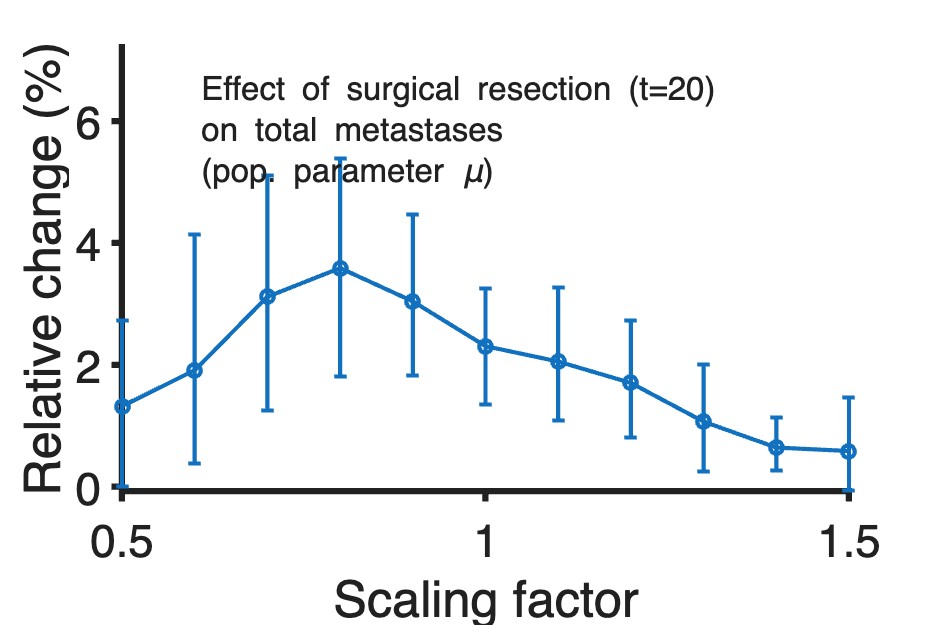

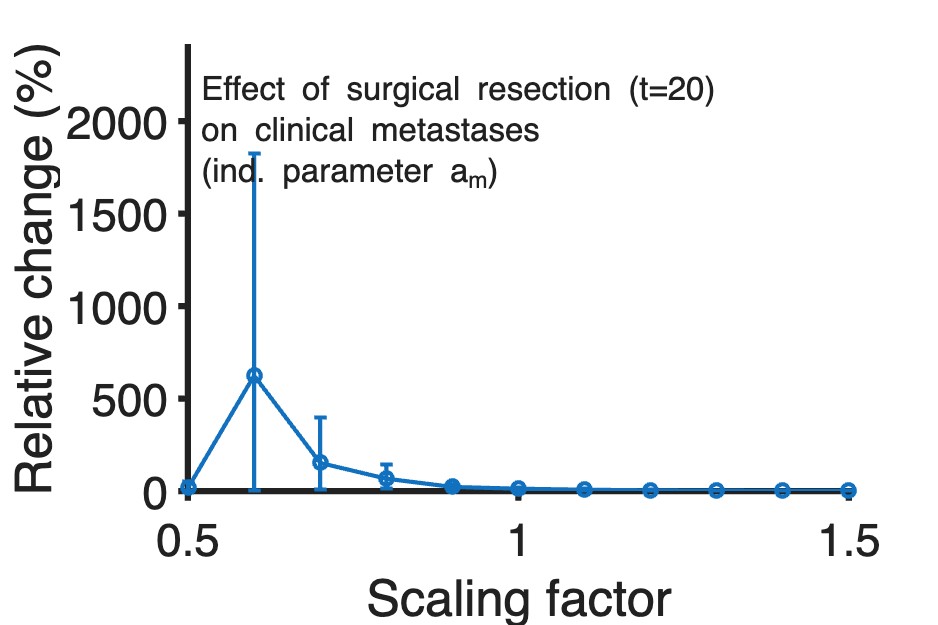

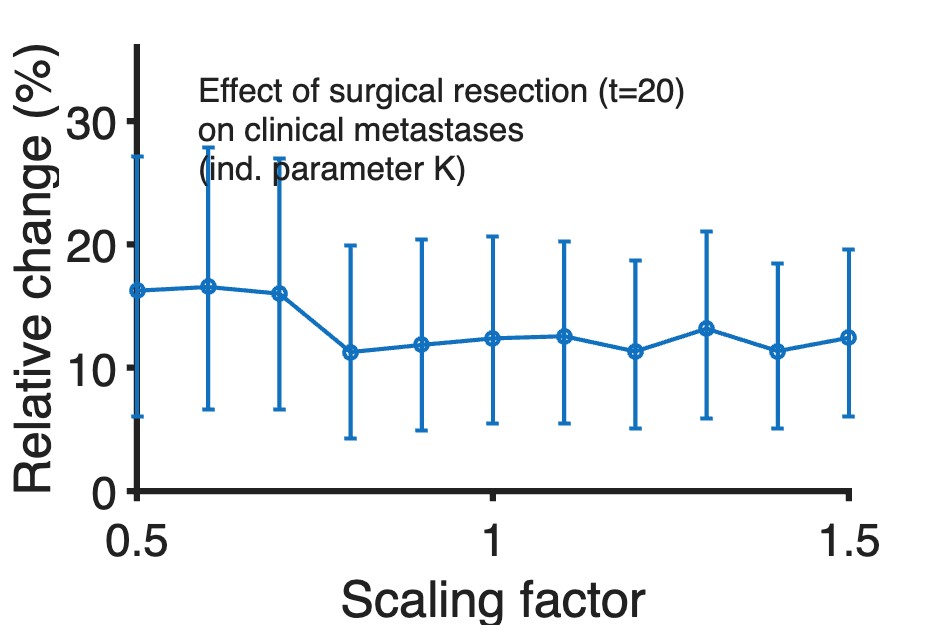


**B**


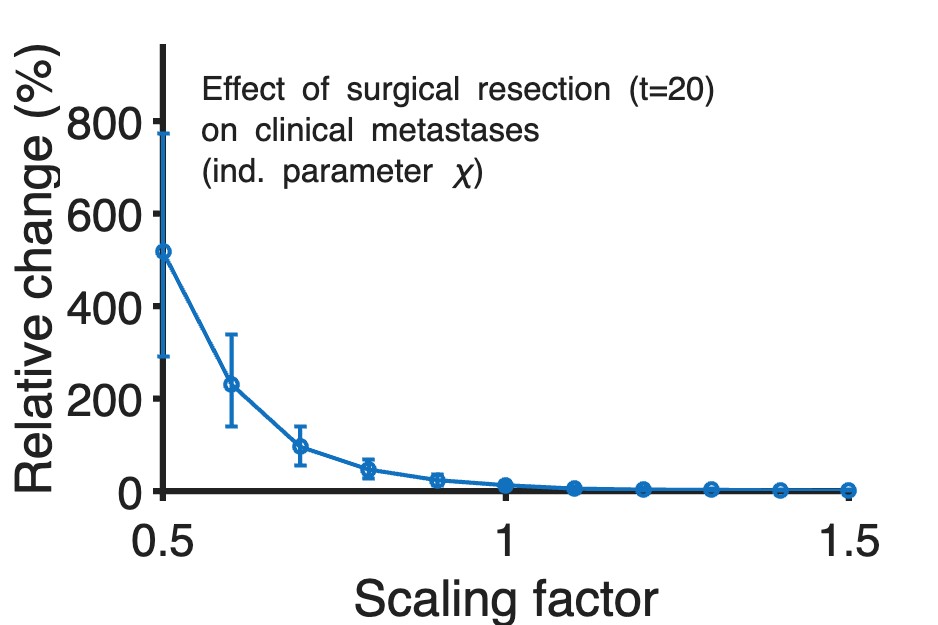

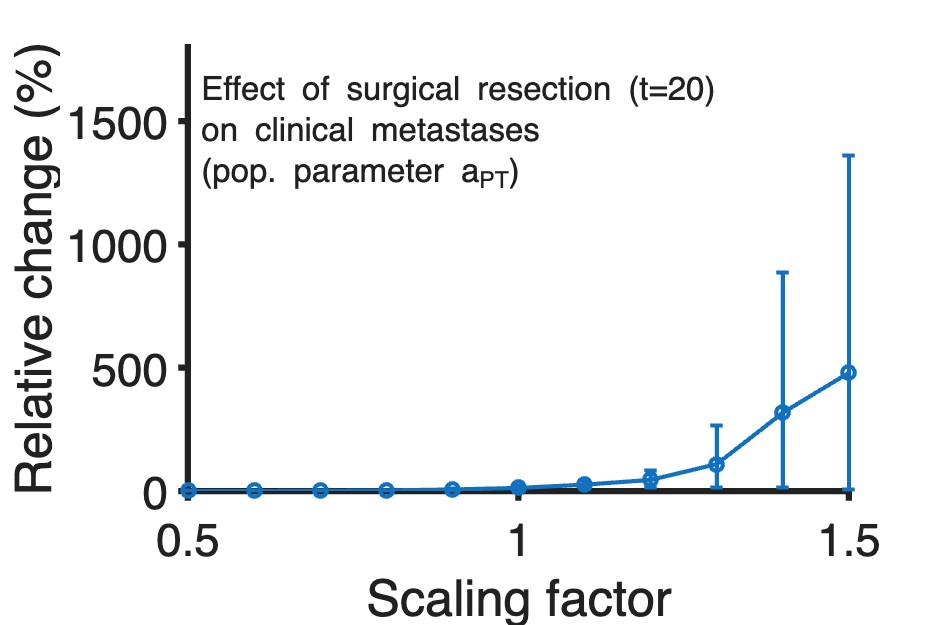

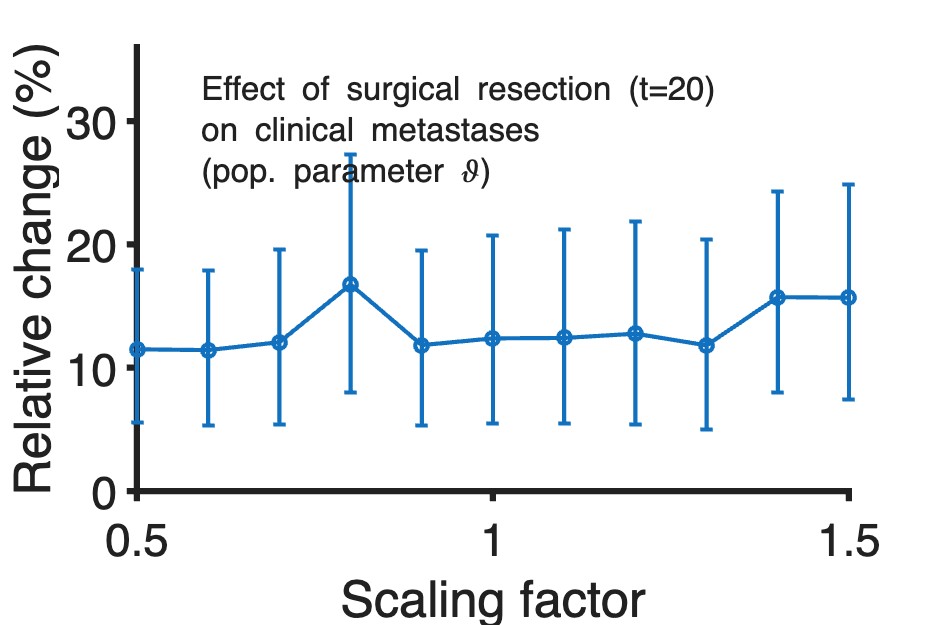

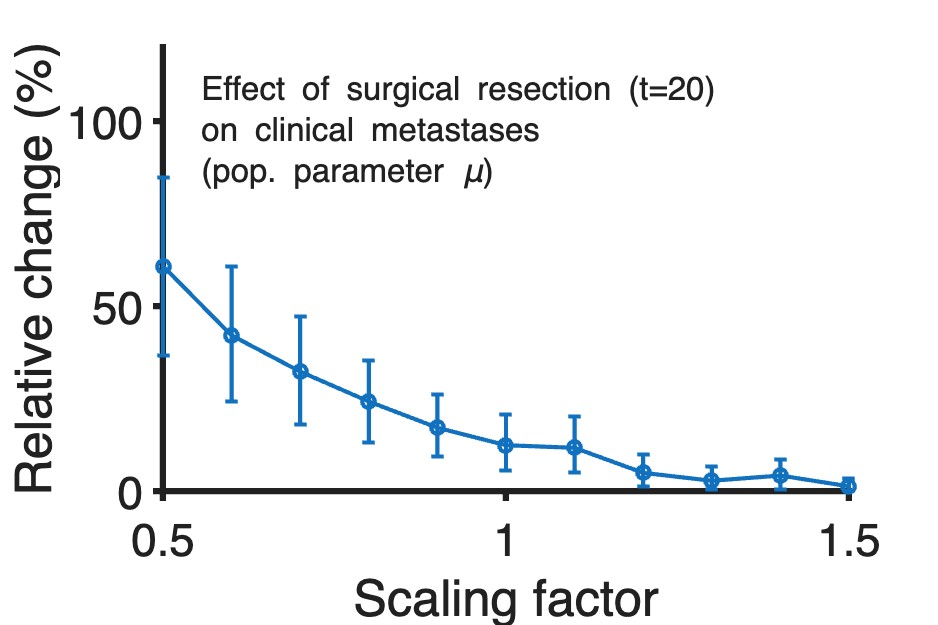


**Figure S10. *Parameter perturbation effects after hypothetical surgery on number of metastases for the* Py230-C57BL/6 group.** These plots show the relative effects of parameter perturbations on the number of **(A)** total and the **(B)** clinically relevant metastases after hypothetical surgery at day 30. For this analysis we neglected mice that did not show metastases at time point of sacrifice.


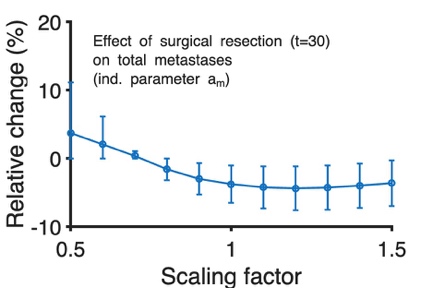

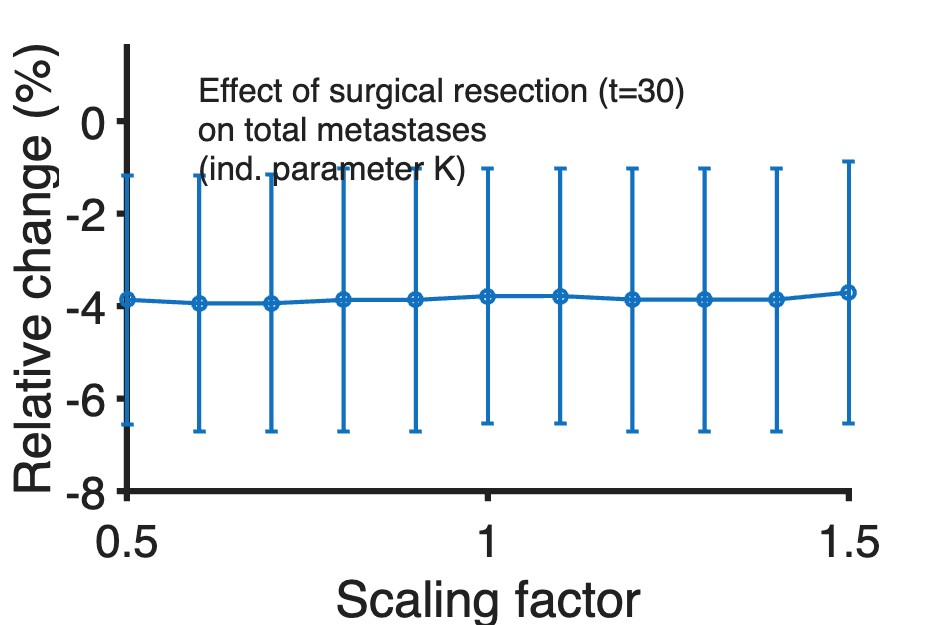


**A**

**B**
